# Dimensions of implementation facilitation (IF): development of a tool for designing, documenting and reporting on IF strategies in healthcare settings

**DOI:** 10.1186/s43058-026-01056-0

**Published:** 2026-07-30

**Authors:** Mona J. Ritchie, Jeffrey L. Smith, Jennifer N. Hill, Katherine M. Dollar, Jeanette A. Waxmonsky, Karen A. Oliver, JoAnn E. Kirchner

**Affiliations:** 1 VA Behavioral Health Quality Enhancement Research Initiative (QUERI), Central Arkansas Veterans Healthcare System, Three Financial Center, 901 South Shackelford Road (5th Floor), Little Rock, AR 72211 USA; 2 Department of Psychiatry, University of Arkansas for Medical Sciences, 4301 W Markham St, #755, Little Rock, AR 72205 USA; 3 Implementation Science Practice, Patient Centered Solutions, IQVIA, 600 Lexington Ave, New York, NY 10022 USA; 4 VA Center for Integrated Healthcare, Syracuse VA Medical Center, 800 Irving Ave, Syracuse, NY 13210 USA; 5 Office of Community Outreach and Engagement, University of Colorado Cancer Center, Anschutz Medical Campus, 13001 E 17th Pl, Aurora, CO 80045 USA; 6 Department of Community and Behavioral Health, Colorado School of Public Health, Anschutz Medical Campus, 13001 E 17th Pl, Aurora, CO 80045 USA; 7 Department of Family Medicine, Anschutz Medical Campus, 12631 E 17th Ave., Aurora, CO 80045 USA; 8 KEAO Consulting, LLC. 2426 1st Ave N, Seattle, WA 98109 USA; 9 School of Pharmacy, Social and Administrative Sciences Division, University of Wisconsin-Madison, 777 Highland Ave, Madison, WI 53705 USA

**Keywords:** Implementation facilitation, Practice facilitation, Implementation strategies, Implementation science

## Abstract

**Background:**

Implementation strategies are operationalized variably and often inadequately described in scientific literature, prompting calls for standardized reporting mechanisms. Implementation facilitation (IF) is a complex, multifaceted, and evidence-based implementation strategy that is one of the more frequently applied and evaluated. Identifying and defining the complete range of IF dimensions is foundational to establishing a more standardized approach to designing, documenting, and reporting on IF strategies. Such work will provide greater transparency and understanding of how the strategy is applied, as well as greater consistency in reporting. This paper describes the development of a tool to support such a standardized approach to IF design, documentation, and reporting in healthcare settings.

**Methods:**

We used a rigorous, multi-phase process to develop and refine a tool to support IF practitioners and researchers in designing, documenting, and reporting on an IF strategy. We validated the tool’s usefulness; verified current reporting of IF dimensions and how they were described in relevant protocol papers; and iteratively refined and enhanced the tool to create the *Implementation Facilitation Dimensions Worksheet*.

**Results:**

We identified and defined thirteen IF dimensions, including Position; IF skills, innovation knowledge, and characteristics; Self-efficacy; Activities; Autonomy/degree of flexibility; Duration; Frequency/intensity; Modes of interaction/mediums used; Role/approach; Change agents; Other stakeholders; Sources of influence; and Resources that support the facilitator. The final publicly available tool includes a structured worksheet that can be used to describe the dimensions of a planned or previously applied IF strategy, a table that lists and defines IF activities that can be used in implementation efforts, instructions and examples for operationalizing the IF dimensions, and a list of other related tools and resources.

**Conclusions:**

*The Implementation Facilitation Dimensions Worksheet* provides guidance and a structured format intended to support the comprehensive design, documentation, and reporting of IF strategies. If the tool achieves its intended purpose, researchers and implementation practitioners may be better informed and equipped to make comparisons of IF strategies across studies and/or to replicate or spread successful IF strategies to other healthcare settings.

**Supplementary Information:**

The online version contains supplementary material available at https://doi.org/10.1186/s43058-026-01056-0.

Contributions to the literature

- Implementation facilitation (IF) is a complex, multifaceted strategy used to implement clinical innovations that has been inconsistently described in the literature. Understanding the complete range of IF dimensions is foundational to establishing a systematic approach to designing, documenting, and reporting the strategy.
- Using a rigorous development process, we identified and described 13 IF dimensions and created a comprehensive tool that provides guidance and structure for designing, documenting, and reporting the dimensions of a particular IF strategy.
- Use of this tool may support standard reporting on IF strategies, support comparisons across studies, and potentially foster replication and spread of those that are successful.

## Background

Implementing evidence-based innovations (EBIs) to improve healthcare delivery and outcomes is often complicated and challenging [1]. The healthcare contexts within which EBIs are implemented are complex and vary in purpose, organizational structure, scope of services, resources available for implementation efforts, patient populations, readiness for change, and many other characteristics [2, 3]. Implementation science is the study of methods and strategies that address these challenges and foster the adoption, implementation, and sustainment of EBIs into routine use [4]. Implementation strategies can be operationalized variably and are often inadequately described in scientific literature [5, 6]. Additionally, most implementation studies use multiple strategies to support EBI implementation, compounding challenges to making comparisons across studies and synthesizing evidence, which hinders efforts to understand how and why strategies are successful [7]. Implementation scientists have thus called for standardized reporting mechanisms to ensure that strategies are clearly labeled and described [6]. For example, in their seminal article, Proctor et al. provide guidance for naming, defining, and specifying the dimensions of implementation strategies [6]. They propose that seven dimensions should be detailed when reporting strategies, including the actor(s), actions, action targets, temporality, dose, implementation outcome affected, and the justification for using the strategy. In addition to calls for standardized reporting, implementation scientists have called for the systematic planning of implementation strategies and the reporting of how they change over time as they are tailored to the local context and its needs [8–10].

Implementation facilitation (IF) [11] is one of the more frequently applied and evaluated implementation strategies [7, 12]. It has been defined as both a role [13], i.e., an individual who conducts IF, and a multi-faceted, interactive process of problem solving, enabling, and supporting efforts to adopt and incorporate innovations into routine practice that occurs in the context of a recognized need for improvement and supportive interpersonal relationships [14, 15]. IF has been characterized by some as a ‘meta-strategy’ [16, 17] because it is typically applied as a primary strategy bundled with multiple other implementation strategies, e.g., audit and feedback, conducting educational meetings, assessing for readiness/identifying barriers, and promoting adaptability within the EBI without compromising fidelity [7, 15]. Adding to the complexity of this strategy, facilitators may be external or internal to the implementation setting [11], and they conduct a wide variety of activities to support implementation [18], tailoring both what they do and implementation of the innovation to the organizational context [19]. To support implementation, facilitators need a diverse set of skills [20], the absence of which can negatively impact the process and outcomes of implementation [21–23]. The length of time facilitators interact with sites, as well as the frequency with which they interact, is also highly variable [24]. Thus, the position of the facilitator in relationship to the setting where implementation occurs, the skills of the facilitator, activities facilitators use, the duration of IF, and the frequency of facilitator interactions with site stakeholders are dimensions of IF that have been inconsistently described in scientific literature. However, given the complexity of the strategy, there are likely other dimensions that are important for successfully supporting EBI implementation.

A growing body of literature supports IF as an evidence-based implementation strategy [7, 12, 25]. IF has improved implementation of EBIs for a wide variety of physical and mental health conditions, i.e., diabetes [26, 27]; kidney disease [28]; cancer [29]; cardiovascular disease [30]; chronic obstructive pulmonary disease [31]; depression [32]; and serious mental illness [33]. IF has also successfully supported implementation of complex care delivery models, e.g., primary care mental health integration [34, 35], the patient centered medical home [36], and collaborative chronic care [37, 38]. It has been tested in a wide variety of healthcare settings, e.g., primary care clinics, mental health clinics, hospitals and pediatric clinics [34, 39–41], including under-resourced settings [34]. Further, one meta-analysis concluded that primary care practices receiving IF were 2.76 times more likely to adopt evidence-based guidelines [42]; and another review found that IF improved outcomes for multiple chronic diseases in primary care [43]. More recent systematic reviews found that IF is among a limited number of implementation strategies with the most robust evidence for effectiveness [7, 12].

Despite ample evidence of IF effectiveness, some studies testing the strategy have not been successful [44–46]. Lack of success has been attributed to factors related to organizational context, e.g., lack of leadership support or staff turnover [23, 47]; characteristics of the innovation, e.g., it was not compatible with practice needs [45]; or the recipients, e.g., clinic staff failed to take advantage of available IF [45]. Facilitators may in some cases not be able to address implementation challenges related to such factors, e.g., when leadership support is needed but lacking and leaders will not adequately engage [44]. However, it could be that failure to adequately consider or address some dimension(s) of IF when designing or applying the strategy may hinder implementation success. Studies have attributed lack of success to characteristics of facilitators, e.g., facilitators’ lack of necessary skills [48] and their personal characteristics and abilities [23], and what facilitators do, e.g., lack of timely responsiveness to partner needs, contributing to poorer relationships [49]; excessive flexibility in terms of facilitators’ latitude in shaping their approach [31]; lack of enough flexibility to overcome the influence of variable contextual factors across settings [46]; or lack of guidance on the structure or content of facilitation visits [31]. Lack of success has also been attributed to how facilitators apply the strategy. For example, the duration or frequency of facilitation was insufficient [50] or facilitators’ contact with sites was irregular [49] or of low intensity [31]. IF studies have also attributed negative results to lack of structural and financial supports for IF [48]. Arguably, these examples of ineffectual IF highlight gaps that might have been addressed through systematic attention to all IF dimensions in designing and/or applying the strategy.

Although scholars have proposed characteristics of contextual, recipient, and innovation factors that influence implementation success [13, 51–53] and identified some key dimensions of IF (e.g., essential skills [20] and core activities [18] involved in applying the strategy), there have to date been no efforts to systematically identify and define or describe the full range of IF dimensions. Identifying and defining the complete range of IF dimensions is foundational to establishing a more systematic approach to comprehensively and systematically describing how IF strategies are, or will be, operationalized so that they can be compared across EBI implementation efforts in healthcare settings [6]. This paper describes the identification of IF dimensions and development of the *Implementation Facilitation Dimensions Worksheet,* a tool for designing, documenting, and reporting of IF strategies. This work was conducted by U.S. Veterans Health Administration (VA) Behavioral Health Quality Enhancement Research Initiative (BH QUERI) investigators and collaborators associated with its activities. This program has been awarded competitive grant funding continuously for over twenty-five years to improve behavioral health care for veterans by developing and evaluating strategies for their effectiveness in implementing EBIs. During the last 15 years, the program has used IF as its primary strategy to support EBI implementation [34, 37, 54, 55] and has specialized in supporting the practice and study of IF by establishing an Implementation Facilitation Learning Collaborative (IFLC) [25]; developing multiple tools, training materials, and other resources that support the practice and evaluation of IF [11, 18, 56–58]; and training and mentoring 700 facilitators at the time of this writing through its Implementation Facilitation Learning Hub [25].

## Methods

We conducted a multi-phase process to develop and refine a tool that may be used to comprehensively design, document, and/or report the dimensions of a planned or applied IF strategy in a standardized manner. In the text that follows, we describe the process for developing the IF Dimensions Worksheet. See Table 1 for the relevant IF experience and qualifications of individuals who led the development process.

**Table 1:** Experience and qualifications of IF dimensions Worksheet developers (*n* = 6)^a^

Collective years of experience	
Serving as a facilitator	102
Evaluating use of IF strategies	100
Developing IF tools, training materials, and other resources	112
Training and/or mentoring facilitators	64
**Scientific contributions related to applying or evaluating IF**	
Number of peer-reviewed publications authored	57
Number of refereed grants as PI, Co-I, or consultant	39

IF: implementation facilitation

^a^ This table summarizes the development team’s (*n* = 6 IF experts) combined years of experience and their scientific contributions in applying or evaluating IF to implement evidence-based innovations in healthcare settings

### Phase 1: Initial development

MJR drafted a preliminary list of eight potential IF dimensions, including facilitation approach, duration. flexibility, frequency and intensity of contact, methods and techniques, mode of interaction/venue, position of facilitator in the organization, and source of influence [6, 59–63]. She presented the list to BH QUERI’s Implementation Facilitation Learning Collaborative (IFLC) [25]. The IFLC consisted of VA operational, programmatic and clinical personnel as well as VA and non-VA researchers interested in advancing the science and practice of IF. Based on IFLC feedback obtained during the meeting, as well as from subsequent conference calls and email correspondence, we further developed the list to include thirteen IF dimensions with definitions and created a worksheet prototype that would allow users to describe or document each dimension of an IF strategy (in a standardized and consistent manner) for a given implementation effort. An IFLC workgroup comprised of MJR, JNH, KMD, JAW, and KAO refined the list of dimensions and definitions and made plans for validating the worksheet’s usefulness and verifying current reporting of IF dimensions to further develop the worksheet.

### Phase 2: Validation and refinement

Next, we validated the worksheet’s usefulness for 1) designing an IF strategy and 2) documenting and reporting on the use of IF in an implementation effort. First, during a 2-day face-to-face meeting to *plan* a research project testing the impact of an IF strategy that would support implementation of measurement-based care (MBC) for mental health in primary care settings [64], the investigative team used the worksheet to design the IF strategy to be applied and tested in the project. The team included MJR, JLS, KMD, and JEK. During the meeting, the team, facilitated by MJR, reviewed the IF dimensions (as described in the worksheet) and planned how each would be operationalized in the project’s IF strategy, e.g., for the *Frequency and intensity* dimension, they identified parameters for how often the facilitator should meet with local quality improvement teams during different phases of the project. After the investigative team completed designing their IF strategy guided by the worksheet, MJR conducted a verbal debriefing with other team members, asking them to provide feedback on the usefulness of the worksheet for designing the IF strategy and took notes to document their responses.

Second, to validate the worksheet’s usefulness for *documenting and reporting* on a strategy, investigators who were presenting seven studies to the IFLC used the worksheet to document the dimensions of the IF strategy they had applied. After using the preliminary thirteen-item worksheet to design an IF strategy and reviewing IFLC presenters’ documentation, we concluded that the worksheet was somewhat cumbersome in presentation and that some of the dimensions seemed to have a similar focus. We applied a commonly-used qualitative analysis technique for grouping conceptually similar items [65; p. 275] to cluster the dimensions into five categories, ‘characteristics of facilitators,’ ‘what facilitators do,’ ‘how facilitators do it,’ ‘with whom facilitators do it,’ and ‘resources that can help facilitators do it.’ We revised the worksheet by organizing the dimensions into these categorical clusters to provide structure and allow users to consider IF dimensions within each area of focus. We then assessed the worksheet’s usability and utility for retrospectively reporting on the dimensions of IF strategies applied in the seven studies. For purposes of this work, “usability” refers to how easy (or complicated) the worksheet was to use by those who completed it; and “utility” refers to the functionality or how well it does the job it was intended to do. Typically, validation is conducted from the user’s perspective. We conducted the validation of the worksheet’s usefulness and functionality from the perspective of experts, assuming that if users understood IF dimension labels and descriptions in the worksheet, they would be able to document the IF strategy they used. Thus, two experts (KD and JEK) reviewed dimension descriptions on each of the 7 completed worksheets and rated presenters’ descriptions of each dimension on a scale of 1 to 4 (1 = presenter did not attempt to describe; 2 = presenter attempted to describe but description was not relevant; 3 = presenter partially, but did not fully, describe the dimension, i.e., something was missing; and 4 = presenter fully described the dimension). The raters also had the option of selecting “TBD - the information is unclear and needs to be discussed.” We compared ratings for each dimension and identified ratings on which they disagreed. Raters reviewed and discussed these and came to consensus on final ratings.

Raters conducted 91 individual ratings of 13 IF dimensions across the 7 studies; 67 of the final ratings (73.6%) indicated that the dimension was fully described. However, only seven of the thirteen dimensions (53.8%) were rated as fully described in at least six of the presentations. We thus asked raters how we might improve the clarity and understandability of dimension labels and definitions on the worksheet, particularly for the dimensions less consistently rated as fully described. In addition to recommendations for improving labels and definitions, raters suggested that we provide users of the worksheet with examples and instructions. We then further revised the worksheet to incorporate the raters’ recommendations.

### Phase 3: Verification and further refinement

To assess current reporting of IF dimensions to inform how they may be operationalized in planned studies, MJR conducted a review of 53 protocol papers for studies using IF strategies published from 2008 to 2023 in the journal, *Implementation Science*. She imported protocol papers into Atlas.ti qualitative data analysis software for coding of text about IF dimensions. For each dimension, she created a summary report that included the number and percentage of papers describing the dimension and a brief summary of what was learned. We then further refined the worksheet and created an appendix (included in the worksheet document) that contains additional information and examples of how IF dimensions were operationalized and reported in the protocol papers.

### Phase 4: Final revisions

**Fig. 1:**
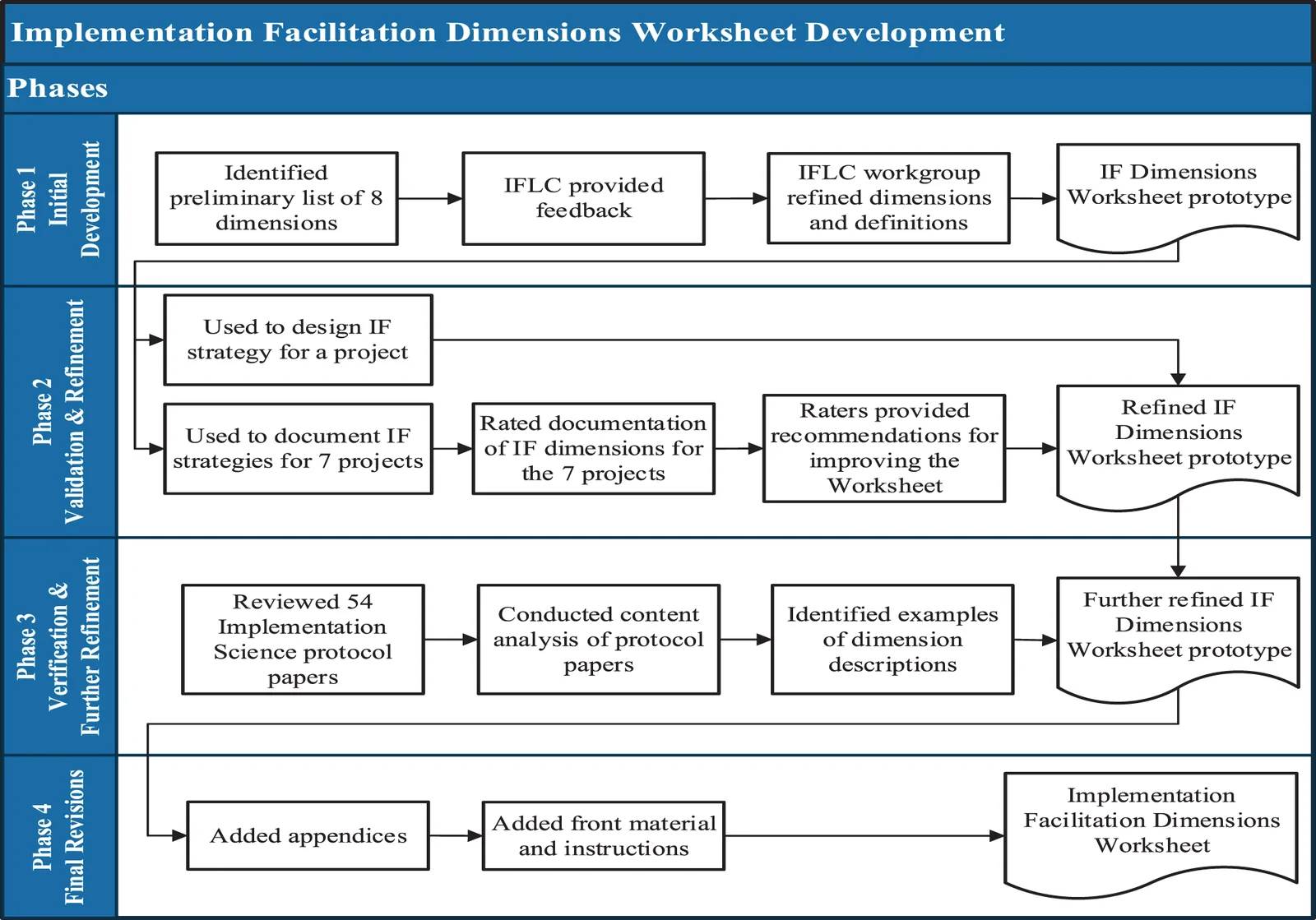
Development process for implementation facilitation (IF) dimensions Worksheet

In this last phase of development, we added two additional appendices to the worksheet and introductory text describing its development and instructions for its use. We then reviewed and made final edits to the worksheet, resulting in a comprehensive tool for use by implementation researchers and practitioners in designing, documenting, and reporting on IF strategies. Figure 1 (below) provides an overview of all 4 phases of the development process.

## Results

We identified and defined 13 dimensions of IF that are included in the Implementation Facilitation Dimensions Worksheet. See Table 2 for the names and final descriptions of the 13 IF dimensions.

**Table 2:** Dimensions of implementation facilitation (IF)

Characteristics of Facilitators	
Position	Defined by location and perhaps by reporting structure. External facilitator(s) are positioned outside of the site or system implementing the innovation. Internal facilitators are located within an implementation site or system.
IF skills, innovation knowledge, and characteristics	Skills needed to apply IF, including stakeholder engagement, problem identification and solving, and assessment skills; credible knowledge about the innovation being implemented; and the facilitator’s personal characteristics (e.g., ability to empathize and understand the needs of others; relevant knowledge, work experience, and other types of training they have received).
Self-efficacy	The facilitator’s confidence in their ability to apply IF.
**What Facilitators Do**	
Activities	Activities facilitators apply to support innovation implementation. ‘Core activities’ have been identified for IF which should usually be applied regardless of the innovation or setting.
Autonomy/degree of flexibility	The degree to which facilitators have autonomy in making decisions about the activities they employ and the scope of their efforts. Although flexibility is a hallmark of facilitation, an IF strategy may be prescriptive, and the facilitator can only be flexible within the structure of the IF strategy design.
**How Facilitators Do It**	
Duration	The length of time facilitators provide support for implementing an innovation.
Frequency and intensity	How often interactions occur (e.g., once a week, bi-weekly, or ×times per month) and the length of time for those interactions (e.g., 30 minutes).
Modes of interaction and mediums used	The way facilitators interact or modes of interaction (e.g., in-person, virtual) and the mediums, venues, and platforms (e.g., Teams, Zoom, WebEx, email, and/or text messaging) facilitators use to communicate with stakeholders.
Role/approach	The purpose of IF and the facilitator’s approach to supporting implementation on a continuum from task-focused to enabling.
**With Whom Facilitators Do It**	
Change agents	Individuals who support implementation at the local level (e.g., champions, opinion leaders and/or quality improvement/implementation teams) or outside the local level (e.g., subject matter experts and technical assistants).
Other stakeholders	Other individuals who can impact implementation and/or who will be directly affected by the implementation effort (e.g., providers, staff, patients, leaders).
**Resources That Can Help Facilitators Do It**	
Sources of influence	Resources that are available for persuading, inspiring, motivating or in some other way affecting change in stakeholders’ perceptions, attitudes, beliefs or behavior.
Resources that support the facilitator	Resources that support facilitators can be human, financial, and/or structural (e.g., salary support, dedicated time for facilitation activities, program support for administrative tasks, IF training).

IF: implementation facilitation

### Description of tool

The final tool (available in Additional File 1 and online at https://www.queri.research.va.gov/tools/implementation.cfm) includes a cover page, the IF Dimensions Worksheet, and 3 appendices. The cover page includes a brief description of IF, a summary of how the worksheet was developed, and instructions for its use. The IF Dimensions Worksheet includes the list of dimensions described above with an expanded description of each dimension. The worksheet can be used to describe the operationalization of a planned or previously applied IF strategy. Appendix A is a table that lists and defines 32 distinct IF activities identified in collateral work by members of this team [18]. Rather than describing the operationalization of the *IF Activities* dimension in the Worksheet, users can simply select and describe the specific facilitator activities for their strategy within this table in Appendix A, if they prefer. Appendix B includes additional examples and information obtained from our review of the *Implementation Science* protocol papers, with citations for specific examples. Appendix C includes additional IF tools and resources that can support users in completing the worksheet as well as in applying and evaluating an IF strategy.

### Usability of Worksheet for designing, documenting, and reporting

Researchers and IF practitioners who used the worksheet to design the IF strategy for the measurement-based care study found it both easy to use and useful for specifying how IF would be operationalized in the study. The IF practitioner identified as the external facilitator for the study found the process of using the Worksheet particularly valuable as it gave him directions for operationalizing his role in supporting the implementation of measurement-based care.

As previously mentioned, 7 of the dimensions were rated as fully described in at least 6 of the 7 IFLC presentations examined. Thus, 6 dimensions were less consistently rated as fully described, including facilitators’ knowledge, characteristics, and skills; facilitators’ self-efficacy; other change agents; resources that support facilitators; modes of contact and venues; and approach. Raters disagreed most on their initial ratings of knowledge, characteristics, and skills, agreeing during the consensus process that 5 of 7 presenters only partially described this dimension. They recommended that the label be changed to IF *Skills, innovation knowledge, and characteristics* and described in a way that emphasizes the importance of all 3 components of this dimension. Ratings of the *Self-efficacy* dimension indicated that this dimension was the least well described; 6 of 7 of the IFLC presentations either lacked any documentation or only partially described the dimension. As a result, raters recommended a complete revision of the *Self-efficacy* dimension’s description. They also recommended that the labels and descriptions be revised for other dimensions, including stakeholders targeted for contact, other change agents, and approach. Although they mostly rated dimensions of *Frequency and intensity* and *Modes of interaction and mediums used* as fully described, raters felt descriptions lacked specificity. Raters struggled with rating several dimensions (e.g., *Sources of influence* and *Resources that support the facilitator*) because they did not have enough information about the project to assess whether presenters had described all of the resources. Finally, in addition to fully describing an IF dimension, presenters sometimes provided additional information that was not relevant for a particular dimension, e.g., by including information about the facilitator’s knowledge and activities in the description of the *Role/approach* dimension.

### Documentation of dimensions in protocol papers

Three (6%) of the 53 protocol papers reviewed described 11–12 of the 13 IF dimensions; and 20 of the papers (38%) described 9–10 dimensions. No protocol papers described all 13 IF dimensions. The percentage of papers in which individual IF dimensions were described was highly variable (see Table 3). The most frequently described dimensions were *IF activities*, *Position* of facilitator, *Duration* of IF, and *Frequency and intensity* of IF. The least frequently described dimensions were *Change agents* who supported implementation, the facilitator’s *Autonomy and degree of flexibility*, *Resources that support facilitators*, and the *Role/approach* facilitators take to supporting implementation. No papers mentioned the *Self-efficacy* of facilitators. See Additional File 2 for results of protocol paper review.

**Table 3:** Mention/Description of IF dimensions in *implementation science* protocol papers reviewed (*n* = 53)

IF Dimension	**N**^**a**^ **(%)**^**b**^ **of papers**
IF Activities	52 (98%)
Position	46 (87%)
Duration	44 (83%)
Frequency and intensity	43 (81%)
Modes of interaction and mediums used	42 (79%)
Sources of influence	40 (76%)
Other stakeholders	38 (72%)
IF skills, innovation knowledge, and characteristics	33 (62%)
Change agents	24 (45%)
Autonomy/degree of flexibility	22 (42%)
Resources that support the facilitator	22 (42%)
Role/approach facilitators take	9 (17%)
Self-efficacy	0 (0%)

IF: implementation facilitation

^a^ *N* = number of *Implementation Science* papers in which IF Dimension was mentioned or described

^b^Denominator = 53 *Implementation Science* protocol papers reviewed

The amount of information describing some of the dimensions was also highly variable in the protocol papers. For example, for the *IF activities* dimension, some papers described IF activities in detail and others provided very brief descriptions. Additionally, dimensions that included multiple components did not always describe all components. For example, only eight papers (15%) described all 3 components of the facilitators’ *IF skills, innovation knowledge, and characteristics* dimension, and only fifteen papers (28%) mentioned anything about the facilitators’ innovation knowledge. For the *Frequency and intensity* dimension, only twelve papers (23%) described both the frequency and intensity of IF.

## Discussion

The *Implementation Facilitation Dimensions Worksheet* was developed to support IF practitioners and researchers in the design, documentation, and reporting of IF. Authors used a rigorous and iterative development process to: a) identify IF dimensions based on literature, feedback from representative end-users, group discussion, and consensus; b) create a worksheet prototype; c) validate the prototype’s usefulness and functionality; d) verify IF dimensions and how they were operationalized in relevant protocol papers; and e) refine and enhance the prototype to create a useful tool. The Worksheet includes instructions and recommendations for designing, documenting, or reporting 13 dimensions of an IF strategy, as well as definitions and examples of each dimension.

Literature suggests that implementation strategies are often operationalized variably and inadequately described [5, 6]; and our review of *Implementation Science* protocol papers confirmed that this is true of IF strategies. None of the IF dimensions we identified were described in all of the protocol papers; 4 of them were described in 45% or less of the protocol papers; and 1 dimension was not described in any of the papers. To validate the Worksheet’s usefulness and functionality, seven IFLC presenters documented the dimensions of the IF strategy they applied to support EBI implementation using an early prototype of the Worksheet. We found that having a structured worksheet for reporting on IF dimensions may be helpful for improving description of the IF strategy used in EBI implementation efforts. For example, the *Autonomy/degree of flexibility, Change agents,* and *Resources that support facilitators* dimensions were described in only 42% to 45% of the protocol papers we reviewed but were fully or partially described on all of the prototype worksheets completed by IFLC presenters. Even the *Self-efficacy* dimension, which was not described in any of the protocol papers, was fully or partially described in most of the prototype worksheets. This is a promising finding in terms of the tool’s potential to support enhanced IF documentation and reporting, as classic learning theory [66, 67] emphasizes the importance of self-efficacy (i.e., confidence in one’s skills and abilities to perform a specific behavior) in learning and applying new behaviors. Given IF’s relative complexity and multifaceted nature, self-efficacy in delivering the strategy may be of particular importance; accordingly, we retained the *Self-efficacy* dimension in the Worksheet even though it was not reported in IF study protocols reviewed. Retention of the *Self-efficacy* dimension in the final Worksheet is also consistent with our IF training program’s evaluation methodology, which routinely assesses pre-/post- changes in trainees’ confidence in applying IF skills [25, 68].

It is possible that a few of the IF dimensions identified in the Worksheet may not be applicable in all IF efforts. For example, some sites may not have local change agents available to assist with implementation efforts; or the facilitator in a completed project may have been an expert or highly experienced, and so self-efficacy may not be a consideration (or even answerable) for post-project reporting.

The IF Dimensions Worksheet may be useful for a variety of purposes. For example, it can be used to prospectively guide the design of an IF strategy (prior to its use in a project) to try and ensure that all dimensions are taken into consideration and documented in the design process. The Worksheet may also be used to support more standardized reporting of IF strategies after studies or initiatives are completed, for greater transparency in describing how IF was applied so successful strategies might be replicated more reliably (e.g., in potential scale-up or spread efforts to follow). Greater standardization in reporting of IF strategies may also provide richer and more complete data for cross-study comparisons, systematic reviews, and/or meta-analyses to examine and explain IF effectiveness and mechanisms thereof. For evaluation purposes in a given project or initiative, the completion of an IF Dimensions Worksheet might contribute data for analyses focused on examining whether/how operationalization of IF dimensions may be associated with implementation success or failure. Worksheet data may be particularly useful in this regard for multi-site projects and/or projects involving use of multiple facilitators, when dimensions related to facilitator characteristics, presence of change agents, mode and frequency of interactions, sources of influence, etc. may vary considerably from facilitator to facilitator and site to site. Finally, to support assessment of IF fidelity in a project or initiative [18], the IF Dimensions Worksheet can be used before projects are initiated to document the *planned* IF strategy (including planned use of core activities [18]) and again at project conclusion to document the IF strategy as actually *applied*. This would allow assessment and critical examination of how the applied IF strategy may have deviated, and in what way(s), from what was initially planned. Further, with documentation of a planned IF strategy in the Dimensions Worksheet serving as a ‘baseline,’ the Framework for Reporting Adaptations and Modifications to Evidence-based Implementation Strategies (FRAME-IS) [69] may be used in a complementary way to document any adaptations or modifications to the strategy during the project. Complementary use of FRAME-IS with the IF Dimensions Worksheet would allow researchers/practitioners to more fully specify the nature of any modifications to a planned IF strategy during the course of a project. Potentially, FRAME-IS’ utility in guiding documentation and examination of modifications to planned IF strategies might be enhanced and possibly more thorough when used in combination with the comprehensive IF Dimensions Worksheet.

The IF Dimensions Worksheet addresses the call for standardizing implementation strategy reporting mechanisms and conforms to the recommendations of Proctor et al. (2013) [6]. Some of the IF dimensions on the Worksheet are similar to the Proctor et al. dimensions. For example, the *Other stakeholders* dimension in the Worksheet is a component of Proctor’s “targets of the action,” and the *Activities* dimension closely matches Proctor’s “actions” dimension. Some of the IF dimensions expand on those recommended by Proctor et al. For example, the Worksheet assumes that the primary “actor” is a facilitator. Because studies have shown that the characteristics of facilitators, e.g., their skills [21, 47], knowledge of the innovation [47], and position in the organization [70] can impact the effectiveness of an IF strategy, the Worksheet includes dimensions intended to guide documentation/reporting of such characteristics. Further, the Worksheet includes a number of dimensions unique to IF which are unrelated to those recommended by Proctor et al., including *Sources of Influence, Resources that support facilitators* (the absence of which can impede facilitation [48]), and the facilitator’s *Role/approach*, a dimension that has been widely described in IF literature [21, 71, 72]. Conversely, there are also several Proctor et al. dimensions that are not represented on the Worksheet, including “implementation outcomes affected” and the “justification” for the strategy. Although these are not included in the Worksheet (given its focus on dimensions for operationalizing an IF strategy), we agree with Proctor et al. that those additional dimensions relevant to *any* implementation strategy should also be reported.

### Strengths and limitations

Strengths of this work include: a) our systematic engagement of IF researchers and practitioners as well as a broader IF learning community [25] in an iterative, multi-phase process to develop, apply, and refine the IF Dimensions Worksheet; b) our application of a Worksheet prototype to document IF strategies used in 7 implementation studies and elicitation of users’ and expert raters’ feedback on the tool’s function and utility to inform refinements; and c) our review of an additional 53 *Implementation Science* protocol papers for studies using an IF strategy in efforts to further validate, refine, and enhance the tool (e.g., by adding an appendix to the tool with examples of how IF dimensions were documented in some of those studies). Additionally, although our work focused on the use of IF for implementing evidence-based innovations in healthcare settings, the tool might be adapted to design and document use of IF to support implementation of practices, programs, or policies in other settings. Such adaptations would obviously warrant further study.

Despite these strengths, there are potential limitations. The Worksheet was only used to support the design of an IF strategy for one project in the development process. While certainly a limitation, we suggest it also represents an *opportunity* for implementation researchers/practitioners to use the tool, critically appraise its utility in addressing *all* of its intended purposes (i.e., support greater standardization and comprehensiveness in design, documentation, and reporting of IF strategies), and propose any refinements that may improve its performance. We will be doing so in our own work going forward and encourage colleagues in the broader field to consider doing the same. Finally, as previously mentioned, the results of our iterative verification and validation processes informed significant revisions to the IF Dimensions Worksheet during its development. Future users should continue to assess its functionality to evaluate its usefulness and/or inform any needs for further refinement.

## Conclusions

Utilizing an iterative multi-phase process, we developed and refined a comprehensive tool, the Implementation Facilitation Dimensions Worksheet, for use in systematically designing, documenting, or reporting on the dimensions of a planned or applied IF strategy to support implementation efforts in healthcare settings. The Worksheet provides a structured format, as well as guidance for its use. If the Worksheet achieves its intended purposes to bring about greater standardization in documenting and reporting IF strategies, researchers and implementation practitioners may be better informed and equipped to make comparisons of IF strategies across studies and/or to replicate or spread successful IF strategies to other healthcare settings. Implementation researchers and practitioners are encouraged to use the tool and critically appraise its utility for accomplishing these and potentially other purposes as well.

## Electronic supplementary material

Below is the link to the electronic supplementary material.

Supplementary Material 1

Supplementary Material 2

## Data Availability

Not applicable.

## Abbreviations

BH QUERI: Behavioral Health Quality Enhancement Research Initiative
EBI: Evidence-based innovation
IF: Implementation facilitation
IFLC: Implementation Facilitation Learning Collaborative
MBC: Measurement based care
VA: U.S. Veterans Health Administration

## References

[1] Silver M, Colditz GA, Emmons KM The promise and challenges of dissemination and implementation research. in: Brownson, RC, Colditz, GA, Proctor, EK, editors. Dissemination and implementation research in health: translating science to practice. 3rd. New York: Oxford University Press; 2023. p. 3–26.

[2] Kitson A, Brook A, Harvey G, Jordan Z, Marshall R, O’Shea RWilson D, et al. Using complexity and network concepts to inform healthcare knowledge translation. Int J Health Policy Manag. 2017;7(3):231–43. 10.15171/ijhpm.2017.79. PMC5890068 29524952

[3] May CR, Johnson M, Finch T. Implementation, context and complexity. Implementation Sci. 2016;11(1):141. 10.1186/s13012-016-0506-3. PMC5069794 27756414

[4] Bauer MS, Damschroder L, Hagedorn H, Smith J, Kilbourne AM. An introduction to implementation science for the non-specialist. BMC Psychol. 2015;3(1):32. 10.1186/s40359-015-0089-9. PMC4573926 26376626

[5] Hooley C, Amano T, Markovitz L, Yaeger L, Proctor E. Assessing implementation strategy reporting in the mental health literature: a narrative review. Adm Policy Ment Health. 2020;47(1):19–35. 10.1007/s10488-019-00965-8. PMC6946851 31482489

[6] Proctor E, Powell B, McMillen J. Implementation strategies: recommendations for specifying and reporting. Implementation Sci. 2013;8(1):139. 10.1186/1748-5908-8-139. PMC3882890 24289295

[7] Ashcraft LE, Goodrich DE, Hero J, Phares A, Bachrach RL, Quinn DA, et al. A systematic review of experimentally tested implementation strategies across health and human service settings: evidence from 2010-2022. Implementation Sci. 2024;19(1):43. 10.1186/s13012-024-01369-5. PMC11194895 38915102

[8] Kirchner JE, Waltz TJ, Powell BJ, Woodward EN, Smith JL, Proctor EK Implementation strategies. in: Brownson, RC, Colditz, GA, Proctor, EK, editors. Dissemination and implementation research in health: translating science to practice. 3rd. New York: Oxford University Press; 2023. p. 119–46.

[9] Fernandez ME, Ten Hoor GA, Van Lieshout S, Rodriguez SA, Beidas RS, Parcel G, et al. Implementation mapping: using intervention mapping to develop implementation strategies. Front Public Health. 2019;7:158. 10.3389/fpubh.2019.00158. PMC6592155 31275915

[10] McHugh SM, Riordan F, Curran GM, Lewis CC, Wolfenden L, Presseau J, et al. Conceptual tensions and practical trade-offs in tailoring implementation interventions. Front.Health Serv. 2022;2:- 2022. 10.3389/frhs.2022.974095. PMC10012756 36925816

[11] Ritchie MJ, Dollar KM, Miller CJ, Smith JL, Oliver KA, Kim B, et al. Using implementation facilitation to improve healthcare (version 3). Veterans Health Administration, Behavioral Health Quality Enhancement Research Initiative (QUERI). 2020. Available from: . https://www.queri.research.va.gov/tools/Facilitation-Manual.pdf

[12] Hero J, Goodrich D, Ernecoff N, Qureshi N, Ashcroft L, Newberry S. Implementation strategies for evidence-based practice in health and health care: a review of the evidence. RAND Health Care, Quality Measurement and Improvement Program & The Veterans Health Foundation. 2023.

[13] Harvey G, Kitson A. Implementing evidence-based practice in healthcare: a facilitation guide. London: Routledge; 2015.

[14] Perry CK, Damschroder LJ, Hemler JR, Woodson TT, Ono SS, Cohen DJ. Specifying and comparing implementation strategies across seven large implementation interventions: a practical application of theory. Implementation Sci. 2019;14(1):32. 10.1186/s13012-019-0876-4. PMC6429753 30898133

[15] Powell BJ, Waltz TJ, Chinman MJ, Damschroder LJ, Smith JL, Matthieu MM, et al. A refined compilation of implementation strategies: results from the expert recommendations for implementing change (ERIC) project. Implementation Sci. 2015;10(1):21. 10.1186/s13012-015-0209-1. PMC4328074 25889199

[16] Kilbourne AM, Geng E, Eshun-Wilson I, Sweeney S, Shelley D, Cohen DJ, et al. How does facilitation in healthcare work? Using mechanism mapping to illuminate the black box of a meta-implementation strategy. Implement Sci Commun. 2023;4(1):53. 10.1186/s43058-023-00435-1. PMC10190070 37194084

[17] Goodrich DE, Miake-Lye I, Braganza MZ, Wawrin N, Kilbourne AM. Quality Enhancement Research Initiative. QUERI roadmap for implementation and quality improvement: United States Department of Veterans Affairs. Veterans Health Administration Office of Research and Development Health Services Research and Development. 2020. Available from: . https://www.queri.research.va.gov/tools/roadmap/33400452

[18] Smith JL, Ritchie MJ, Kim B, Miller CJ, Chinman MJ, Adam Kelly P, et al. Getting to fidelity: consensus development process to identify core activities of implementation facilitation. Glob Implement Res Appl. 2024;4(2):151–66. 10.1007/s43477-024-00119-5. PMC11100021 38765294

[19] Nguyen AM, Cuthel A, Padgett DK, Niles P, Rogers E, Pham-Singer H, et al. How practice facilitation strategies differ by practice context. J Gen Intern Med. 2020;35(3):824–31. 10.1007/s11606-019-05350-7. PMC7080927 31637651

[20] Ritchie MJ, Parker LE, Kirchner JE. From novice to expert: a qualitative study of implementation facilitation skills. Implement Sci Commun. 2020;1(1):7. 10.1186/s43058-020-00006-8. PMC7427882 32885184

[21] Due TD, Thorsen T, Waldorff FB, Kousgaard MB. Role enactment of facilitation in primary care – a qualitative study. BMC Health Serv Res. 2017;17(1):593. 10.1186/s12913-017-2537-0. PMC5569467 28835276

[22] Baloh J, Zhu X, Ward MM. Types of internal facilitation activities in hospitals implementing evidence-based interventions. Health Care Manage Rev. 2018;43(3):229–37. 10.1097/hmr.0000000000000145. PMC6816731 28125454

[23] Rycroft-Malone J, Seers K, Eldh AC, Cox K, Crichton N, Harvey G, et al. A realist process evaluation within the Facilitating Implementation of Research Evidence (FIRE) cluster randomised controlled international trial: an exemplar. Implementation Sci. 2018;13(1):138. 10.1186/s13012-018-0811-0 PMC6238283 30442165

[24] Fasugba O, Cheng H, Dale S, Coughlan K, McInnes E, Cadilhac DA, et al. Finding the right dose: a scoping review examining facilitation as an implementation strategy for evidence-based stroke care. Implementation Sci. 2025;20(1):4. 10.1186/s13012-025-01415-w. PMC11731140 39806380

[25] Kirchner JE, Dollar KM, Smith JL, Pitcock JA, Curtis ND, Morris KK, et al. Development and preliminary evaluation of an implementation facilitation training program. Implement Res Pract. 2022;3:26334895221087475. 10.1177/26334895221087475. PMC9924286 37091085

[26] Dickinson WP, Dickinson LM, Nutting PA, Emsermann CB, Tutt B, Crabtree BF, et al. Practice facilitation to improve diabetes care in primary care: a report from the EPIC randomized clinical trial. Ann Fam Med. 2014;12(1):8–16. 10.1370/afm.1591. PMC3896533 24445098

[27] Grunfeld E, Manca D, Moineddin R, Thorpe KE, Hoch JS, Campbell-Scherer D, et al. Improving chronic disease prevention and screening in primary care: results of the BETTER pragmatic cluster randomized controlled trial. BMC Fam Pract. 2013;14(1):175. 10.1186/1471-2296-14-175. PMC4225577 24252125

[28] Fox CH, Vest BM, Kahn LS, Dickinson LM, Fang H, Pace W, et al. Improving evidence-based primary care for chronic kidney disease: study protocol for a cluster randomized control trial for translating evidence into practice (TRANSLATE CKD). Implementation Sci. 2013;8(1):88. 10.1186/1748-5908-8-88. PMC3751479 23927603

[29] Aspy CB, Enright M, Halstead L, Mold JW. Improving mammography screening using best practices and practice enhancement assistants: an Oklahoma Physicians Resource/Research Network (OKPRN) study. J Am Board Fam Med. 2008;21(4):326. 10.3122/jabfm.2008.04.070060 18612059

[30] Liddy C, Hogg W, Russell G, Wells G, Armstrong CD, Akbari A, et al. Improved delivery of cardiovascular care (IDOCC) through outreach facilitation: study protocol and implementation details of a cluster randomized controlled trial in primary care. Implementation Sci. 2011;6(1):110. 10.1186/1748-5908-6-110. PMC3197547 21952084

[31] Due TD, Thorsen T, Kousgaard MB, Siersma VD, Waldorff FB. The effectiveness of a semi-tailored facilitator-based intervention to optimise chronic care management in general practice: a stepped-wedge randomised controlled trial. BMC Fam Pract. 2014;15(1):65. 10.1186/1471-2296-15-65. PMC4234362 24716545

[32] Fortney J, Enderle M, McDougall S, Clothier J, Otero J, Altman L, et al. Implementation outcomes of evidence-based quality improvement for depression in VA community based outpatient clinics. Implementation Sci. 2012;7(1):30. 10.1186/1748-5908-7-30. PMC3353178 22494428

[33] Kilbourne AM, Abraham K, Goodrich D, Bowersox N, Almirall D, Lai Z, et al. Cluster randomized adaptive implementation trial comparing a standard versus enhanced implementation intervention to improve uptake of an effective re-engagement program for patients with serious mental illness. Implementation Sci. 2013;8(1):136. 10.1186/1748-5908-8-136. PMC3874628 24252648

[34] Kirchner JE, Ritchie MJ, Pitcock JA, Parker LE, Curran GM, Fortney JC. Outcomes of a partnered facilitation strategy to implement primary care–mental health. J Gen Intern Med. 2014;29(S4):904–12. 10.1007/s11606-014-3027-2. PMC4239280 25355087

[35] Ritchie MJ, Parker LE, Edlund CN, Kirchner JE. Using implementation facilitation to foster clinical practice quality and adherence to evidence in challenged settings: a qualitative study. BMC Health Serv Res. 2017;17(1):294. 10.1186/s12913-017-2217-0. PMC5397744 28424052

[36] Nutting PA, Crabtree BF, Stewart EE, Miller WL, Palmer RF, Stange KC, et al. Effect of facilitation on practice outcomes in the National Demonstration Project model of the patient-centered medical home. Ann Fam Med. 2010;8(Suppl Suppl_1):S33–44. 10.1370/afm.1119 PMC2885723 20530393

[37] Bauer MS, Miller CJ, Kim B, Lew R, Stolzmann K, Sullivan J, et al. Effectiveness of implementing a collaborative chronic care model for clinician teams on patient outcomes and health status in mental health: a randomized clinical trial. JAMA Netw Open. 2019;2(3):e190230–e. 10.1001/jamanetworkopen.2019.0230. PMC6484628 30821830

[38] Kilbourne AM, Goodrich DE, Lai Z, Almirall D, Nord KM, Bowersox NW, et al. Reengaging veterans with serious mental illness into care: preliminary results from a national randomized trial. Psychiatr Serv. 2015;66(1):90–93. 10.1176/appi.ps.201300497. PMC4640185 25554233

[39] Mignogna J, Hundt NE, Kauth MR, Kunik ME, Sorocco KH, Naik AD, et al. Implementing brief cognitive behavioral therapy in primary care: a pilot study. Behav. Med. Pract. Policy Res. 2014;4(2):175–83. 10.1007/s13142-013-0248-6. PMC4041920 24904701

[40] Ayieko P, Ntoburi S, Wagai J, Opondo C, Opiyo N, Migiro S, et al. A multifaceted intervention to implement guidelines and improve admission paediatric care in Kenyan district hospitals: a cluster randomised trial. PLoS Med. 2011;8(4):e1001018. 10.1371/journal.pmed.1001018. 10.1371/journal.pmed.1001018 PMC3071366 21483712

[41] Baum RA, Hoholik S, Maciejewski H, Ramtekkar U. Using practice facilitation to improve depression management in rural pediatric primary care practices. Pediatr Qual Saf. 2020;5(3):e295. 10.1097/pq9.0000000000000295. PMC7297391 32656464

[42] Baskerville NB, Liddy C, Hogg W. Systematic review and meta-analysis of practice facilitation within primary care settings. Ann Fam Med. 2012;10(1):63–74. 10.1370/afm.1312. PMC3262473 22230833

[43] Wang A, Pollack T, Kadziel LA, Ross SM, McHugh M, Jordan N, et al. Impact of practice facilitation in primary care on chronic disease care processes and outcomes: a systematic review. J Gen Intern Med. 2018;33(11):1968–77. 10.1007/s11606-018-4581-9. PMC6206351 30066117

[44] Kinley J, Stone L, Dewey M, Levy J, Stewart R, McCrone P, et al. The effect of using high facilitation when implementing the Gold Standards Framework in Care Homes programme: a cluster randomised controlled trial. Palliat Med. 2014;28(9):1099–109. 10.1177/0269216314539785 24942282

[45] Gold R, Bunce A, Cowburn S, Davis JV, Nelson JC, Nelson CA, et al. Does increased implementation support improve community clinics’ guideline-concordant care? results of a mixed methods, pragmatic comparative effectiveness trial. Implementation Sci. 2019;14(1):100. 10.1186/s13012-019-0948-5. PMC6894475 31805968

[46] Seers K, Rycroft-Malone J, Cox K, Crichton N, Edwards RT, Eldh AC, et al. Facilitating implementation of research evidence (FIRE): an international cluster randomised controlled trial to evaluate two models of facilitation informed by the Promoting Action on Research Implementation in Health Services (PARIHS) framework. Implementation Sci. 2018;13(1):137. 10.1186/s13012-018-0831-9 PMC6238407 30442174

[47] Harvey G, McCormack B, Kitson A, Lynch E, Titchen A. Designing and implementing two facilitation interventions within the ‘Facilitating Implementation of Research Evidence (FIRE)’ study: a qualitative analysis from an external facilitators’ perspective. Implementation Sci. 2018;13(1):141. 10.1186/s13012-018-0812-z PMC6238352 30442157

[48] Lizarondo L, McArthur A, Porche D, Corremans M, Perrenoud B, Rodrigues R, et al. Facilitation in evidence implementation – experiences, challenges, and determinants of perceived effectiveness: a qualitative systematic review. JBI Evidence Implementation. 2023;21(4):409–31. 10.1097/xeb.0000000000000399. 37975298

[49] Hogg W, Baskerville N, Nykiforuk C, Mallen D. Improved preventive care in family practices with outreach facilitation: understanding success and failure. J Health Serv Res Policy. 2002;7(4):195–201. 10.1258/135581902320432714. 12425778

[50] Williams L, Daggett V, Slaven JE, Yu Z, Sager D, Myers J, et al. A cluster-randomised quality improvement study to improve two inpatient stroke quality indicators. BMJ Qual Saf. 2016;25(4):257. 10.1136/bmjqs-2015-004188. 26303644

[51] Damschroder LJ, Aron DC, Keith RE, Kirsh SR, Alexander JA, Lowery JC. Fostering implementation of health services research findings into practice: a consolidated framework for advancing implementation science. Implementation Sci. 2009;4(1):50. 10.1186/1748-5908-4-50. PMC2736161 19664226

[52] Damschroder LJ, Reardon CM, Widerquist MAO, Lowery JC. The updated Consolidated Framework for Implementation Research based on user feedback. Implementation Sci. 2022;17(1). 10.1186/s13012-022-01245-0 PMC9617234 36309746

[53] Nilsen P, Bernhardsson S. Context matters in implementation science: a scoping review of determinant frameworks that describe contextual determinants for implementation outcomes. BMC Health Serv Res. 2019;19(1):189. 10.1186/s12913-019-4015-3. PMC6432749 30909897

[54] Wray LO, Oslin DW, Leong SH, Pitcock JA, Tauriello S, Drummond KL, et al. Enhancing implementation of measurement-based mental health care in primary care: a mixed-methods study. Psychiatr Serv. 2023;74(7):746–55. 10.1176/appi.ps.20220140. 36597698

[55] Chinman M, Goldberg R, Daniels K, Muralidharan A, Smith J, McCarthy S, et al. Implementation of peer specialist services in VA primary care: a cluster randomized trial on the impact of external facilitation. Implementation Sci. 2021;16(1):60. 10.1186/s13012-021-01130-2. PMC8183089 34099004

[56] Ritchie MJ, Kirchner JE, Townsend JC, Pitcock JA, Dollar KM, Liu C-F. Time and organizational cost for facilitating implementation of primary care mental health integration. J Gen Intern Med. 2020;35(4):1001–10. 10.1007/s11606-019-05537-y. PMC7174254 31792866

[57] Ritchie MJ, Drummond KL, Smith BN, Sullivan JL, Landes SJ. Development of a qualitative data analysis codebook informed by the i-PARIHS framework. Implement Sci Commun. 2022;3(1):98. 10.1186/s43058-022-00344-9. PMC9476709 36104801

[58] Ritchie MJ, Smith JL, Kim B, Woodward EN, Kirchner JE. Building a sharable literature collection to advance the science and practice of implementation facilitation. Front Health Serv. 2024;4:- 2024. 10.3389/frhs.2024.1304694. PMC11111980 38784706

[59] Harvey G, Fitzgerald L, Fielden S, McBride A, Waterman H, Bamford D, et al. The NIHR Collaboration for Leadership in Applied Health Research and Care (CLAHRC) for Greater Manchester: combining empirical, theoretical and experiential evidence to design and evaluate a large-scale implementation strategy. Implementation Sci. 2011;6(1):1–12. 10.1186/1748-5908-6-96 PMC3170237 21861886

[60] Stetler CB, Legro MW, Rycroft-Malone J, Bowman C, Curran G, Guihan M, et al. Role of “external facilitation” in implementation of research findings: a qualitative evaluation of facilitation experiences in the veterans health administration. Implementation Sci. 2006;1(1):23. 10.1186/1748-5908-1-23. PMC1635058 17049080

[61] Dogherty EJ, Harrison MB, Graham ID. Facilitation as a role and process in achieving evidence-based practice in nursing: a focused review of concept and meaning. Worldviews Evidence-Based Nurs. 2010;7(2):76–89. 10.1111/j.1741-6787.2010.00186.x. 20180826

[62] Dogherty EJ, Harrison MB, Baker C, Graham ID. Following a natural experiment of guideline adaptation and early implementation: a mixed-methods study of facilitation. Implementation Sci. 2012;7(1):9. 10.1186/1748-5908-7-9. PMC3296591 22309743

[63] Liddy C, Hogg W, Singh J, Taljaard M, Russell G, Deri Armstrong C, et al. A real-world stepped wedge cluster randomized trial of practice facilitation to improve cardiovascular care. Implementation Sci. 2015;10(1):150. 10.1186/s13012-015-0341-y. PMC4625868 26510577

[64] Wray LO, Ritchie MJ, Oslin DW, Beehler GP. Enhancing implementation of measurement-based mental health care in primary care: a mixed-methods randomized effectiveness evaluation of implementation facilitation. BMC Health Serv Res. 2018;18(1):753. 10.1186/s12913-018-3493-z. PMC6171308 30285718

[65] Miles MB, Huberman AM, Saldaña J. Qualitative data analysis: a methods sourcebook. Thousand Oaks, CA: Sage; 2014.

[66] Bandura A. Self-efficacy: the exercise of control. New York: W.H. Freeman and Company; 1997.

[67] Mantey DS, Hunt ET, Hoelscher DM, Kelder SH. Social cognitive theory and health behavior. In: Glanz K, Rimer BK, Viswanath K, editors. Health behavior theory, research, and practice. 6th ed. New Jersey: John Wiley & Sons, Inc; 2024. p. 98–115.

[68] Smith JK, Dollar KM, Pitcock JA, Martin JN, Shook CB, Woodward EN, et al. editors. Sustained effectiveness of training program to transfer implementation facilitation knowledge and skills. AcademyHealth 18th Annual Conference on the Science of Dissemination & Implementation. National Harbor, MD: 2025.

[69] Miller CJ, Barnett ML, Baumann AA, Gutner CA, Wiltsey-Stirman S. The FRAME-IS: a framework for documenting modifications to implementation strategies in healthcare. Implementation Sci. 2021;16(1):36. 10.1186/s13012-021-01105-3. PMC8024675 33827716

[70] Smith SN, Liebrecht CM, Bauer MS, Kilbourne AM. Comparative effectiveness of external vs blended facilitation on collaborative care model implementation in slow-implementer community practices. Health Serv Res. 2020;55(6):954–65. 10.1111/1475-6773.13583. PMC7704469 33125166

[71] Harvey G, Loftus-Hills A, Rycroft-Malone J, Titchen A, Kitson A, McCormack B, et al. Getting evidence into practice: the role and function of facilitation. J Adv Nurs. 2002;37(6):577–88. 10.1046/j.1365-2648.2002.02126.x. 11879422

[72] Ritchie MJ, Parker LE, Kirchner JE. Facilitating implementation of primary care mental health over time and across organizational contexts: a qualitative study of role and process. BMC Health Serv Res. 2023;23(1):565. 10.1186/s12913-023-09598-y. PMC10233920 37259064

