# Supplementary material for: Dimensions of implementation facilitation (IF): development of a tool for designing, documenting and reporting on IF strategies in healthcare settings
Source: Implement Sci Commun. 2026 Jul 30;7:212. doi: 10.1186/s43058-026-01056-0 (PMC13711840; doi:10.1186/s43058-026-01056-0)
Supplement: Supplementary file 1 [file 43058_2026_1056_MOESM1_ESM.docx]

**Implementation Facilitation (IF) Dimensions Worksheet:**

**A Tool for Designing, Documenting and Reporting on IF Strategies**

Implementation strategies are methods used for enhancing adoption, implementation, sustainment and scale-up of innovations (e.g., evidence-based practices and other new programs or practices) [1,2]. Implementation facilitation (IF) is a multifaceted implementation strategy involving an interactive process of problem-solving, enabling, and supporting individuals in their efforts to adopt and incorporate clinical innovations into routine practice that occurs in the context of a recognized need for improvement and supportive interpersonal relationships [3,4]. IF, which has been widely used in healthcare settings, is multidimensional and complex, typically bundling an integrated set of activities to address challenges or barriers in supporting innovation implementation [5]. Similar to other implementation strategies, IF has been inconsistently labeled and described in the literature making it difficult at times to understand how the strategy was actually applied in a given implementation effort, which can make it challenging to replicate and/or compare to other studies using IF. Implementation scientists have long agreed on the importance of using standardized approaches for reporting and specifying the dimensions of implementation strategies [1]. Although scholars have identified some dimensions of IF (e.g., activities facilitators conduct, duration and frequency of interactions), little attention has been focused on ensuring a more standardized, comprehensive approach for documenting the dimensions of IF. For purposes of this tool, ‘*dimensions*’ refer to fundamental aspects or features of an IF strategy that should be clearly described and documented to provide transparency on how it was operationalized so it can be reliably replicated, evaluated, and/or compared to other strategies [1].

**About the Worksheet**

This tool was initially developed by VA Behavioral Health Quality Enhancement Research Initiative (BH QUERI) implementation scientists, based on IF literature and input from members of BH QUERI’s Implementation Facilitation Learning Collaborative, which consists of VA operational, programmatic, and clinical personnel, as well as VA and non-VA researchers and implementation practitioners. Modifications were made to the original tool prototype after verifying its utility for designing and reporting on IF strategies. Further modifications were made to reflect the latest IF research and to add examples of dimensions based on a review of *Implementation Science* protocol papers for studies applying IF. Guidance in this Worksheet is consistent with Proctor et al. recommendations for specifying and documenting implementation strategies [1].

This tool identifies and describes 13 dimensions of IF and provides a standardized structure for planning, documenting and retrospectively reporting on the strategy for implementation efforts conducted in healthcare settings. The Worksheet lists the IF dimensions with questions and additional information on each dimension to help guide you in: (a) prospectively designing the IF strategy for a particular implementation effort, (b) documenting adaptations to the planned IF strategy during the implementation effort, and/or (c) retrospectively documenting the IF strategy used at the conclusion of an implementation effort. The Worksheet also includes three appendices to assist you in completing it for your implementation effort. Appendix A provides a comprehensive list of IF activities to help guide your selection or documentation of activities for Dimension #4 (Activities) on the Worksheet. Appendix B provides examples of how IF dimensions have been documented in protocol papers published in the journal *Implementation Science*. Appendix C lists resources where you may find additional information about IF.

**Instructions for using the Worksheet**

For each dimension of your IF strategy, review the question and description on the Worksheet, as well as examples in Appendix B. Then, document on the Worksheet how you plan to address (or addressed) that dimension for the IF strategy to be used (or used) in your implementation effort. Provide this documentation in the column labeled description of your IF strategy. Although some of the questions about the dimensions assume a single facilitator, please be sure to document information about all of the facilitators involved in your implementation effort as their personal characteristics will vary, and what they do in a given project may vary as well. For example, in a project using both external and internal facilitators, the characteristics and activities of those facilitators may vary in important ways. For Dimension #4 (Activities), you may document the IF activities to be used (or used) in your implementation effort by checking them off in the Appendix A table and, if needed, making notes in that table’s “Additional Description” column.

**Implementation Facilitation (IF) Dimensions Worksheet**

| **Dimensions**  (see Appendix B for additional examples and information) | **Description of Your IF Strategy** |
| --- | --- |
| **Characteristics of Facilitators** | |
| 1) **Position**   What is the planned (or what was the) position of the facilitator in relationship to the organization?  *Position is defined by location and perhaps by reporting structure. External facilitator(s) are positioned outside of the site or system implementing the innovation. Internal facilitators are located within an implementation site or system. An IF strategy may include either an external or internal facilitator, or a combination of both an external and internal facilitator(s).* |  |
| 2) **IF skills, innovation knowledge, and characteristics**   What are / were the facilitator’s IF skills, knowledge of the innovation, and personal characteristics?  *Scholars agree that facilitators need a wide range of skills to apply IF, including communication, interpersonal, stakeholder engagement, problem identification and solving, and assessment skills [6,7]. However, the types of skills they need may depend on characteristics of the innovation being implemented, recipients of the innovation, and of the organizational context for change. The experience of the facilitator (e.g., length of time serving in the role, number of implementation efforts in which they have served as a facilitator) and/or the IF training they received may provide some indication of IF skill level.*  *Facilitators also need credible knowledge about the innovation being implemented. If the facilitator is not an expert in the innovation being implemented and/or has no experience in delivering it, he or she should make themselves well enough informed about the innovation and how it works to be able to answer most questions to support its implementation. In such cases, the facilitator should also have ready access to a subject matter expert who can provide answers to stakeholders on more challenging questions pertaining to the innovation (see Dimension #10 below).*  *In addition to IF knowledge and skills, the facilitator’s personal characteristics might impact the facilitation process, e.g.: ability to empathize and understand the needs of others; being credible, approachable, and persistent; relevant knowledge, work experience, and other types of training they have received [8].* |  |
| 3) **Self-efficacy**   How confident is / was the facilitator in their ability to apply IF at the beginning of the project?  *Self-efficacy is the facilitator’s confidence in their ability to apply IF. It may change over time, based on the facilitator’s training and experience. For example, newly trained facilitators (novices) may initially feel confident in their ability but may feel either more or less confident over time based on their experiences in applying the strategy. This dimension might be assessed using a 4-point Likert scale:  (0=Not At All, 1=Somewhat, 2=Moderately, and 3=Extremely).* |  |
| **What Facilitators Do** | |
| 4) **Activities**   What are the planned activities the facilitator will utilize to support implementation (or what were the activities they utilized)?  *Facilitators typically conduct a wide variety of activities to support innovation implementation. An IF strategy may include ‘prescribed’ activities expected to be applied in most/all sites and/or activities may be selected as needed during the facilitation process. ‘Core activities’ have been identified for IF which should usually be applied regardless of the innovation or setting and can be used to assess and ensure fidelity to the IF strategy [9].* | *Go to* *Appendix A* *and select the activities the facilitator will or did use in the implementation effort.* |
| 5) **Autonomy/degree of flexibility**   To what degree does / did the facilitator have autonomy and flexibility to make decisions about their activities and the scope of their work?  *Although flexibility is a hallmark of facilitation, an IF strategy may be prescriptive and the facilitator can only be flexible within the structure of the IF strategy design. This dimension of an IF strategy focuses on the degree to which facilitators have autonomy in making decisions about the activities they employ and the scope of their efforts.* |  |
| **How Facilitators Do It** | |
| 6) **Duration**   What is / was the duration of the IF strategy?  *The length of time facilitators provide support for implementing an innovation is highly variable and may be measured in months or even years. When planning or reporting on an IF strategy, consider whether the duration will be (or was) prescribed or prescribed but then extended. Additionally, consider when during the implementation process facilitation will be (or was) provided (e.g., pre-implementation – 3 months, implementation – 6 months, sustainment – 6 months). For more information about phases of the implementation process, see the introduction to Appendix A and/or Ritchie et al., pages 18-19 [7].* |  |
| 7) **Frequency and intensity**   What is / was the frequency and intensity of the facilitator’s interaction with stakeholders?  *The frequency of interactions (i.e., how often they occur) may be pre-determined (e.g., once a week, bi-weekly, or x times per month), as may the intensity (i.e., length of time or average length of time for those interactions, e.g., 30-minute or hour-long calls). Alternatively, the planned or applied frequency/intensity of interactions may vary based on the needs and characteristics of a given site or may be a mix of regularly scheduled and/or unplanned interactions as needed.* |  |
| 8) **Modes of interaction and mediums used**   What modes of interaction and mediums will / did the facilitator use?  *Facilitators may use a variety of modes of interaction (e.g., in-person, virtual) and mediums, venues, and platforms (e.g., Teams, Zoom, WebEx, email, and text) to communicate with site stakeholders depending on the resources available and the location of the facilitator in relationship to the site. For example, the facilitator may meet with stakeholders in person at the site or may meet with them virtually via phone conferencing, videoconferencing, webinars, or other technology-based mediums.* |  |
| 9) **Role / approach**   What is / was the facilitator’s role and approach (on a continuum from task-focused to enabling) to supporting implementation?  *Although the role (or purpose) of IF generally is to support implementation of an innovation, the role of the facilitator may be unique to a particular facilitation effort. Additionally, the facilitator’s approach to supporting implementation can be described on a continuum from task-focused (e.g., completing specific tasks to support implementation of a particular evidence-based practice or program (EBP)) to enabling (e.g., to develop and empower individuals/teams and create a supportive context for change, not necessarily related to implementing a particular EBP). The facilitator’s approach can lie anywhere along this continuum.* |  |
| **With Whom Facilitators Do It** | |
| 10) **Change agents**   If applicable, who are / were the internal or external change agents working with the facilitator to support implementation?  *Implementation is likely to be more successful when there are internal/local change agents (e.g., champions, opinion leaders and/or quality improvement/implementation teams) engaged in supporting implementation with other stakeholders (see below).*  *Additionally, other external change agents (e.g., subject matter experts, particularly when the facilitator’s experience with the innovation is limited, and technical assistants supported at the system level) may also be engaged in supporting implementation*. |  |
| 11) **Other stakeholders**   Who are / were the other stakeholders the facilitator will engage / engaged to support implementation?  *Facilitators need to engage stakeholders at any organizational level who can impact implementation of the innovation and/or who will be directly affected by the implementation effort. Stakeholders may include providers of the innovation, other providers and staff who refer patients to innovation providers or those who must be involved in the implementation effort for change to occur, patients receiving the innovation, and organizational leaders.* |  |
| **Resources That Can Help Facilitators Do It** | |
| 12) **Sources of influence**   What are / were the sources of influence that the facilitator can use / used to support implementation?  *Sources of influence are resources that are available for persuading, inspiring, motivating or in some other way affecting change in stakeholders’ perceptions, attitudes, beliefs or behavior. Examples of such resources include leadership support for implementation, a policy directive mandating change, research evidence, clinical practice guidelines, facility or unit goals or focus, educational materials, and clinical dashboards.* |  |
| 13) **Resources that support the facilitator**   What resources are / were available to support the facilitator?  *Resources that support facilitators can be human, financial, and/or structural. Examples of such resources include salary support, dedicated time for facilitation activities, program support for administrative tasks, IF training, designated space, and tools for planning/applying IF (e.g., templates (blueprints, playbooks, etc.) for guiding implementation planning with stakeholders; tracking logs for documenting IF time/activities, implementation progress, next steps, etc.).* |  |

**Worksheet References**

1. Proctor EK, Powell BJ, McMillen JC. Implementation strategies: recommendations for specifying and reporting. Implement Sci. 2013;8:1-11.
2. Kirchner JE, Waltz TJ, Powell BJ, Woodward EN, Smith JL, Proctor EK. Implementation strategies. In RC Brownson, GA Colditz, & EK Proctor (eds.), *Dissemination and implemlementation research in health: Translating science to practice (3^rd^ ed.,pp. 119-46). Pxford University Press. 2023*
3. Perry CK, Damschroder LJ, Hemler JR, Woodson TT, Ono SS, Cohen DJ. Specifying and comparing implementation strategies across seven large implementation interventions: a practical application of theory. Implement Sci. 2019;14(1):32.
4. Powell BJ, Waltz TJ, Chinman MJ, et al. A refined compilation of implementation strategies: results from the Expert Recommendations for Implementing Change (ERIC) project. Implement Sci. 2015;10(1):21.
5. Ritchie MJ, Parker LE, Kirchner JE. Facilitating implementation of primary care mental health over time and across organizational contexts: a qualitative study of role and process. BMC Health Serv Res. 2023;23(1):565.
6. Ritchie MJ, Parker LE, Kirchner JE. From novice to expert: a qualitative study of implementation facilitation skills. Implement Sci Commun. 2020;1(1):7.
7. Ritchie MJ, Dollar KM, Miller CJ, et al. Using implementation facilitation to improve healthcare (Version 3): Veterans Health Administration, Behavioral Health Quality Enhancement Research Initiative (QUERI); 2020. Available from: https://www.queri.research.va.gov/tools/Facilitation-Manual.pdf.
8. Elledge C, Avworo A, Cochetti J, Carvalho C, Grota P. Characteristics of facilitators in knowledge translation: an integrative review. Collegian. 2019;26(1):171-82.
9. Smith JL, Ritchie MJ, Kim B, et al. Getting to fidelity: Consensus development process to identify core activities of implementation facilitation. Glob Implement Res Appl. 2024;4(2):151-66.

# Appendix A: Implementation Facilitation Activities to be considered for Dimension #4

The table in this appendix lists and defines 32 distinct IF activities identified through a scoping literature review of 135 articles from 94 IF studies (see: Smith JL, et al., *Global Implementation Research and Applications* 2024;4(2):151-66). For Dimension #4 (Activities), review this list and select the activities used (or to be used) in your IF strategy, paying special attention to ensuring that you use core IF activities (as described below), as well as other activities as needed to support implementation.

*Core IF activities,* i.e., those proposed to be fundamental to maintaining fidelity to an IF strategy, are **bolded** in the table. Using a rigorous consensus development process, these core activities were identified by IF experts as most critical for a facilitator to apply to support implementation of a clinical innovation across three phases of implementation (i.e., Pre-Implementation, Implementation, and Sustainment phases) (see: Smith JL, et al., 2024).

- In the ‘Pre-Implementation’ phase a facilitator engages with sites prior to implementation to gain a better understanding of their resources, priorities and context… with the primary objective being to assist them with developing a tailored, local implementation plan. ***In the table, a superscripted ^‘1’^ identifies the 8 core IF activities for the Pre-Implementation phase.***
- In the ‘Implementation’ phase when the local implementation plan is carried out, the facilitator maintains ongoing relatively frequent contact with sites to monitor and support the plan’s execution and refinement. ***A superscripted ^‘2’^ in the table identifies the 8 core IF activities for the Implementation phase.***
- In the ‘Sustainment’ phase, the facilitator’s contact with sites becomes less frequent and eventually non-existent, with the objective being to turn a mature (hopefully successful) implementation effort over to the site with minimal further interaction. ***A superscripted ^‘3’^ in the table identifies the 4 core IF activities for the Sustainment phase.***

| **Select** | **IF Activities (*core activities in bold)** | **Additional Description** |
| --- | --- | --- |
| **Building relationships, teams, and networks** | | |
|  | ***Engaging stakeholders, obtaining buy-in^1^**  *Engaging relevant stakeholders and seeking their participation/buy-in; building relationships with stakeholders; helping stakeholders ‘own’ the change.* |  |
|  | ***Managing group/team processes^2^**  *Includes managing group dynamics, running effective meetings, keeping group focused, establishing team structure, membership, roles and ground rules, creating atmosphere of mutual respect, enhancing communication, building relationships among (not with) team members, empowering group members, fostering democratic/participatory process.* |  |
|  | Fostering networking with experts  *Linking to external expert contacts; fostering contact with external people/organizations (non-peers) who can provide needed information or services.* |  |
|  | Fostering peer networking  *Fostering networking, communication across or within sites, learning collaboratives / communities of practice; cross-pollination using opinion leaders.* |  |
|  | Overcoming resistance to change  *Working with leaders and providers (including innovation providers) to overcome resistance to innovation. This activity focuses more on addressing individual attitudes than shared beliefs among members of an organization or sub-groups within an organization.* |  |
| **Collecting data / providing feedback** | | |
|  | ***Conduct ongoing monitoring of program implementation^2,3^**  *Monitoring/tracking/collecting data/information on progress, problems/barriers, enablers (facilitators), fidelity to evidence, performance, and innovation activities and linking implementation to outcomes. Includes quantitative and qualitative data, observations, etc.* |  |
|  | ***Data collection to assess context and baseline performance^1^**  *Collecting/reviewing quantitative/qualitative diagnostic information/data to understand the local context, baseline performance, determinants of current practice (barriers and enablers (facilitators)).* |  |
|  | ***Providing updates and feedback^2,3^**  *Providing updates on implementation, including providing feedback on data, PDSA cycles (CQI), innovation provider activities, facilitator activities, relevant professional or system-level information (e.g., availability of new guidelines, tools, awareness of new administrative/clinical policy, etc.). Includes interpretation of data for stakeholders.* |  |
| **Enabling/fostering change** | | |
|  | ***Fostering organizational change: structural^2^**  *Promoting structural change required for implementation (e.g., staffing changes, reporting structure changes, office assignment changes, methods for referring patients, how patients move through the system [e.g., physically get to innovation providers, get from innovation providers to front desk] etc.).* |  |
|  | Fostering organizational change: cultural  *Promoting cultural change required for implementation. Includes changes in shared beliefs about care, types of providers’ skills and practice scopes (not specific providers, but classes of providers such as primary care providers, social workers, psychiatrists, psychologists, nurses, etc.), efficacy and safety of treatments and practice models. These may be shared beliefs among all members of an organization or members of a sub-group (e.g., mental health providers, nursing, primary care providers, physicians etc.).* |  |
|  | Fostering change, unspecified  *Promoting change generally to support implementation, unspecified.* |  |
|  | Interceding and liaising with others  *Interceding/liaising with leadership (internal or external) and/or relevant programs/departments (internal or external) about the innovation (including regulatory issues, facilitation implementation, other issues) and/or stakeholder needs.* |  |
|  | Strategy/policy development  *Assisting with strategy and policy development* |  |
| **Helping to define, identify, and fill stakeholder roles** | | |
|  | ***Describing/clarifying roles and responsibilities^1^**  *Describing purpose and process of innovation, facilitation, what will occur, establishing/clarifying and allocating roles and responsibilities.* |  |
|  | ***Identification/selection of local change agents^1^**  *Helping identify/select, and/or hire local change agents, e.g., internal facilitators, opinion leaders, champions, and quality improvement (QI) team members.* |  |
|  | Helping to hire clinical program staff  *Assisting with hiring innovation providers, innovation provider replacements or additions; includes helping with writing job descriptions.* |  |
| **Disseminating innovation or facilitation knowledge** | | |
|  | ***Action/implementation planning^1^**  *Assisting with the development and refinement of Action Plans / Implementation Plans, including formal action items, short-term plans and long-term plans; assisting sites with an implementation checklist.* |  |
|  | ***Adapting program to local context without compromising fidelity^2^**  *Help to adapt to and create synergy with local context, including local structure, staffing, culture and other initiatives.* |  |
|  | ***Goal/priority setting^1^**  *Assisting in setting clear, realistic goals; setting priorities. Includes assisting with the selection of an area for change and developing/refining specific clinical practice questions.* |  |
|  | Attending, presenting at and/or organizing non-local meetings  *Organizing, attending and/or presenting at regional/national meetings, councils. advisory boards or other forums (not site visits).* |  |
|  | Fostering spread of clinical innovation/facilitation methods  *Assisting with spread of clinical innovation and/or facilitation methods beyond facilitation sites.* |  |
|  | Developing shared vision / consensus building  *Finding synergy between existing goals and innovation goals; helping stakeholders to “get on the same page”; helping stakeholders to see what is in it for “me and us”; developing “win-win solutions”.* |  |
| **Problem identification and resolution** | | |
|  | ***Problem identification^1^**  *Assisting with problem identification, awareness and clarification, including understanding current ways of working and thinking, identifying gaps and barriers in current context.* |  |
|  | ***Problem-solving^2^**  *Assistance with problem-solving, brainstorming solutions.* |  |
| **Providing administrative/technical support** | | |
|  | ***Administrative tasks^1,2^**  *Set up site visits; scheduling and inviting stakeholders to calls and meetings; organizing meetings; emailing announcements and reminders; setting up monthly innovation provider calls; creating and sending out meeting minutes; preparing and disseminating reports and materials; scheduling/arranging speakers for monthly calls; making arrangements for innovation provider training (e.g., sending innovation providers to other clinics to shadow another innovation provider) (Note that only the arrangement part is administrative, not actual provision of training); preparing PowerPoint slides. Does not include organizing regional or national meetings.* |  |
|  | Technical support  *Providing technical support (e.g., providing tools and sample materials [e.g., reminder systems, templates, patient materials, etc.]); developing necessary infrastructure to support adoption other than information technology (IT); assisting with conducting literature searches and appraising and summarizing the evidence; working with stakeholders to create documents/evaluation materials. May also include identifying or providing information about other available resources, such as resources available in community. Also working with IT or other stakeholders to ensure that IT systems accurately capture and support innovation activity (e.g., encounters) or anything related to computer support issues including software.* |  |
| **Providing education/information** | | |
|  | Clinical skills education  *Providing education/information about clinical content skills/expertise, including provider education, clinical training/supervision/coaching and mentoring, academic detailing (can be ongoing). Also includes counseling, co-counseling, critical reflection, new ways of work, experiential learning, learning from practice. Does not include presentations at regional and or national meetings.* |  |
|  | Marketing  *Promoting or publicizing the innovation and what it does, including provision of evidence for innovation conducted by facilitators, highlighting a need for practice change, emphasizing enhanced patient outcomes as reason for change, and interpreting the research for practical application.* |  |
|  | Marketing education  *Education about how to conduct marketing to clinic providers, staff or leadership. Includes teaching innovation providers how to market their own skills and services* |  |
|  | Organizational change skills education  *Providing education/information about change skills/processes, including skills, incentives and resources to implement change and information about organizational systems and change processes, evaluation, how to engage stakeholders in change process, empowering participants.* |  |
| **Using interpersonal skills to create a supportive environment** | | |
|  | ***Providing support^2,3^**  *Being generally helpful and available; communicating regularly; being available for questions; providing encouragement; doing things in a warm, encouraging, and empathetic way rather than hypercritical, punishing way; demonstrating “people skills”; using carrots rather than sticks; acknowledging ideas and efforts and celebrating achievements/success; maintaining momentum and enthusiasm; creating an open, supportive, and trusting environment conducive to change; providing ongoing support/reassurance and constructive feedback. May also include self-disclosure (sharing personal insights or experiences), interjecting humor.* |  |
|  | ***Pulling back and letting sites take lead^3^**  *Pulling back and letting sites take lead in implementation / sustainability* |  |

# Appendix B. Additional Information and Examples of IF Strategy Dimensions from Literature

The following examples and additional information were obtained from our review of all protocol papers using IF as an implementation strategy that were published in *Implementation Science* from 2008 – 2023.

| **Dimensions** | **Additional Information and Examples** |
| --- | --- |
| **Characteristics of Facilitators** | |
| 1) **Position** | **External facilitators** may be the researchers or staff for a particular study or implementation effort. They may be hired by a regional organization (e.g., a practice-based research network [1]; a health management organization [2]; or an AHRQ-funded health care cooperative [3]). Alternatively, they may be employed by a national organization (e.g., in one study, external facilitation would be provided by the VA’s Office of Women’s Health Services [4].  **Internal facilitators** may be organization staff at any level (front-line, managers/supervisors, leadership) who might be selected based on their expertise/experience or their role in the organization.  **Models:** Although an IF strategy may include an EF or an IF or both, some strategies are more complex. For example, in one study, there was going to be an external expert facilitator who would prepare and support multiple internal facilitators (hospital clinical leaders); they, in turn, would support ward staff who would provide internal facilitation at the ward level [5]. Additionally, facilitation may be provided by individuals or teams. For example, one study planned to have an external facilitation team (consisting of clinical and implementation experts) who would work with an internal facilitation team (consisting of an internal facilitator and a champion) [6]. |
| 2) **Skills, knowledge, characteristics** | **IF skills**  Given the wide variety and complexity of IF skills, rather than listing all of the skills, you might describe which of the following IF competencies (or clusters of skills) facilitator(s) need or had: (1) building relationships and creating a supportive environment, (2) changing the system of care and the structure and processes that support it, (3) transferring knowledge and skills and creating infrastructure support for ongoing learning, (4) planning and leading change efforts, and (5) assessing people, processes, and outcomes and creating infrastructure for program monitoring [7].  If internal or external facilitators have not previously conducted IF, how they will be (or were) trained on IF can be described. For example, they might be trained individually or in a group by an IF expert for a particular implementation effort [6] or they may have attended a formal IF training program [4,8]. It may be important to describe components of the training, e.g., evidence presentation, use of implementation strategies, quality improvement methods [9].  **Innovation knowledge**  When selecting facilitator(s), consider what kind of expertise about the innovation they need to support implementation. For example, they may have read about the innovation and studied its implementation; they may have experience in delivering the innovation; or they may have experience in delivering it and training others to deliver it. If they do not have experience delivering the innovation, consider what type of subject matter expert is needed to support implementation, e.g., one that has expertise in the delivery of the innovation or in some technical component of the innovation.  **Characteristics of facilitator(s)**  Personal characteristics of the facilitator(s) might include: 1) personality traits; 2) their occupation, training, and credentials (e.g., in one study, facilitators were health professionals with master’s degrees and clinical and managerial experience in primary care [8]); 3) expertise (e.g., in one study, facilitators would have expertise in social determinants of health, clinic workflows and practice change implementation, and electronic health record use [10]); 4) work experience (e.g., facilitators in one study would be nurses with an average of 35 years in clinical practice [11]); and 5) knowledge (e.g., in one study, facilitators needed to have knowledge of good practice in continence care, co-workers, the environment, and the organization [12]). |
| 3) **Self-efficacy** | Bandura [13] theorized that self-efficacy plays a significant role in influencing how people behave, make decisions, react to changes, cope with challenges, and accomplish things; and extensive research has shown that self-efficacy beliefs influence performance in work settings [14]. Thus, a facilitator’s confidence in their ability to apply IF will likely impact the facilitation process and may impact the implementation process. However, none of the reviewed protocol papers described facilitator’s self-efficacy. Despite the lack of documentation in these papers, when planning or evaluating IF, consider assessing and documenting this dimension of IF. |
| **What Facilitators Do** | |
| 4) **Activities** | Although the list in Appendix A is comprehensive, the description of what facilitators do or did should be related to the innovation and context. In the reviewed protocol papers, there was wide variability in the amount of information describing IF activities. Some papers just listed planned activities (e.g., “stakeholder engagement, education, collaborative problem solving and goal setting, increased direct communication, and ongoing support” [15]. Others provided detailed descriptions of planned IF activities [6,16]. |
| 5) **Autonomy/degree of flexibility** | A study to improve adoption of a program to address obesity prevention in childhood planned to use both a low-intensity and a high-intensity IF strategy. For the low-intensity strategy, facilitators would be confined to using a particular set of activities [17]. However, few protocol papers described facilitator’s autonomy and degree of flexibility. Despite the lack of documentation in these papers, when planning or evaluating IF, consider specifying the autonomy or degree of flexibility that will be or was allowed of facilitators. |
| **How Facilitators Do It** | |
| 6) **Duration** | No additional information or examples (see Worksheet). |
| 7) **Frequency and intensity** | This dimension of IF has important implications for planning and budgeting for an IF strategy, as well as for understanding its effectiveness and budget impact after the fact. However, it was described in varying detail in the reviewed protocol papers. Some papers just described the planned interaction as “regular” and/or with no information about the intensity of the interaction. Some papers reported only interaction frequency. For example, “….once every other week for three to six months, and then monthly over a period of 12 months…practice facilitator will remain available to each practice for ad hoc consultation between team meetings during this 12- month period” [18]. Other papers provided detail about frequency and intensity. For example, “…. approximately 1 h/month in calls with each study clinic, 2 h/ month on office hours, 1–2 h/week to discuss the clinics’ progress and needs internally, and 1–2 h/week to respond to clinic emails, for a total of 15–23 h per month to support five clinics.” Given that the needs of varying contexts often influence the frequency and intensity of facilitator contact across sites, you might consider specifying (before the IF strategy is initiated) minimum and/or maximum levels of facilitator contact planned for sites (which can also inform budget planning). For example, one protocol paper stated, “Practices will receive up to 8 h a month of practice facilitation depending on their level of readiness at enrollment… Practice facilitators (PFs) will record monthly the frequency, duration, and mode of practice contact” [3]. In addition, during an implementation effort or at its end, you may find that ‘what was planned’ in terms of frequency/intensity of facilitator contact with participating sites can differ considerably from ‘what is/was actually done’. Thus, consider using some form of the ‘IF Time and Activity Tracking Tool’ (see Appendix C) that facilitators can use to document the frequency and intensity of their contacts with sites. For documentation purposes on *this* Worksheet, such tracking tool data could be used to document the ‘range’ of frequency/intensity of facilitator contact observed in the implementation effort (which can inform budget planning for subsequent (spread) efforts to implement the innovation in new sites). |
| 8) **Modes of interaction and/or mediums used** | No additional information or examples (see Worksheet). |
| 9) **Role / approach** | The facilitators’ roles in the majority of reviewed protocols were primarily task-focused, i.e., helping sites implement a particular innovation. Several of the reviewed protocol papers planned to compare a more task focused IF model with an enabling one. For example, one study will compare the effect of a low-intensity task-focused IF strategy vs. a high-intensity enabling, holistic IF strategy on fidelity to an intervention to increase children’s intake of carotenoid-rich fruits and vegetables and child health outcomes [17]. For the task-focused strategy, facilitators will “monitor implementation, identify and solve problems related to contextual barriers, and assist with navigating structural changes needed for [the innovation].” For the enabling, holistic strategy, facilitators will “support educators in a one-on-one fashion, helping to set goals, fostering peer networking, developing shared vision among leaders and staff, and building meaningful relationships that support change efficacy.” Most protocol papers did not describe this dimension explicitly but focused on supporting implementation of a particular innovation rather than building capacity for the process of implementation. |
| **With Whom Facilitators Do It** | |
| 10) **Change agents** | No additional examples. Facilitators may only work with other change agents, e.g., champions {Gold2019} or implementation teams [19], who also support implementation. Alternatively, facilitators may work with both change agents and other stakeholders. For example, Owen et al. worked with clinic champions (internal change agents) and clinical providers and leadership (other stakeholders) [20]. |
| 11) **Other stakeholders** | No additional examples. Although facilitators may work with both change agents and other stakeholders, for some implementation efforts, they may only work with stakeholders who would not be considered change agents (e.g., all mental health providers [21]). |
| **Resources That Can Help Facilitators Do It** | |
| 12) **Sources of influence** | Additional examples of sources of influence that facilitators may use include: leveraging relationships with a large healthcare system’s national program offices to engage site leaders [22]; leveraging organizational or governmental policies (e.g., a mandate to implement a national suicide risk identification strategy [15], a Nigerian Federal Ministry of Health policy [11]; educational toolkits and materials (e.g., sample scripts, testimonials, motivational interview examples [17]; systems engineering tools [19]; and data dashboards [23]). |
| 13) **Resources that support the facilitator** | Examples of funding resources include: sponsored research projects; external agencies (e.g., a population-based health management organization [24]; responsibility for facilitation as part of a funded organizational role (e.g., Intimate partner violence (IPV) Coordinators at VA facilities were funded by Congress [4]; or protected time for facilitators (e.g., one study will provide payment to meet the costs of covering shifts for internal facilitators [12].  Examples of training/toolkits: formal IF training programs (e.g., State University of New York at Buffalo Practice Facilitator Certificate Program [25], training program led by expert facilitators [6], participation in shared learning opportunities with other facilitators [11], or mentoring [e.g., [17]). Examples of educational materials and other tools for supporting facilitators include facilitation guides [9], meeting agendas and worksheets [26], and outcome reports [27].  Examples of consultation and other types of support: In one study, practice facilitation managers will meet weekly with groups of facilitators to provide oversight and shared learning opportunities [25]. In another study, national operational leaders in VA’s IPV Assistance Program will provide consultation [4]. Feedback on audits of the implementation strategy and fidelity will be provided to internal facilitators in another study [28]. |

**Appendix B** **References**

1.	Tapp H, McWilliams A, Ludden T, et al. Comparing traditional and participatory dissemination of a shared decision making intervention (ADAPT-NC): a cluster randomized trial. Implement Sci. 2014;9(1):158.

2.	Roberge P, Fournier L, Brouillet H, et al. Implementing a knowledge application program for anxiety and depression in community-based primary mental health care: a multiple case study research protocol. Implement Sci. 2013;8(1):26.

3.	Weiner BJ, Pignone MP, DuBard CA, et al. Advancing heart health in North Carolina primary care: the Heart Health NOW study protocol. Implement Sci. 2015;10(1):160.

4.	Iverson KM, Dichter ME, Stolzmann K, et al. Assessing the Veterans Health Administration’s response to intimate partner violence among women: protocol for a randomized hybrid type 2 implementation-effectiveness trial. Implement Sci. 2020;15(1):29.

5.	Aifah AA, Odubela O, Rakhra A, et al. Integration of a task strengthening strategy for hypertension management into HIV care in Nigeria: a cluster randomized controlled trial study protocol. Implement Sci. 2021;16(1):96.

6.	Midboe AM, Martino S, Krein SL, et al. Testing implementation facilitation of a primary care-based collaborative care clinical program using a hybrid type III interrupted time series design: a study protocol. Implement Sci. 2018;13(1):145.

7.	Ritchie MJ, Parker LE, Kirchner JE. From novice to expert: a qualitative study of implementation facilitation skills. Implement Sci Commun. 2020;1(1):7.

8.	Liddy C, Hogg W, Russell G, et al. Improved delivery of cardiovascular care (IDOCC) through outreach facilitation: study protocol and implementation details of a cluster randomized controlled trial in primary care. Implement Sci. 2011;6(1):110.

9.	Malone S, McKay VR, Krucylak C, et al. A cluster randomized stepped-wedge trial to de-implement unnecessary post-operative antibiotics in children: the optimizing perioperative antibiotic in children (OPerAtiC) trial. Implement Sci. 2021;16(1):29.

10.	Gold R, Bunce A, Cowburn S, et al. Does increased implementation support improve community clinics’ guideline-concordant care? Results of a mixed methods, pragmatic comparative effectiveness trial. Implement Sci. 2019;14(1):100.

11.	Aifah AA, Hade EM, Colvin C, et al. Study design and protocol of a stepped wedge cluster randomized trial using a practical implementation strategy as a model for hypertension-HIV integration — the MAP-IT trial. Implement Sci. 2023;18(1):14.

12.	Seers K, Cox K, Crichton NJ, et al. FIRE (facilitating implementation of research evidence): a study protocol. Implement Sci. 2012;7(1):25.

13.	Bandura A. Self-efficacy: Toward a unifying theory of behavioral change. Psychol Rev. 1977;84(2):191-215.

14.	Bandura A, Locke EA. Negative self-efficacy and goal effects revisited. J Appl Psychol. 2003;88(1):87-99.

15.	Bahraini NH, Matarazzo BB, Barry CN, et al. Protocol: examining the effectiveness of an adaptive implementation intervention to improve uptake of the VA suicide risk identification strategy: a sequential multiple assignment randomized trial. Implement Sci. 2020;15(1):58.

16.	Kilbourne AM, Abraham K, Goodrich D, et al. Cluster randomized adaptive implementation trial comparing a standard versus enhanced implementation intervention to improve uptake of an effective re-engagement program for patients with serious mental illness. Implement Sci. 2013;8(1):136.

17.	Swindle T, Rutledge JM, Selig JP, et al. Obesity prevention practices in early care and education settings: an adaptive implementation trial. Implement Sci. 2022;17(1):25.

18.	Parchman ML, Pugh JA, Culler SD, et al. A group randomized trial of a complexity-based organizational intervention to improve risk factors for diabetes complications in primary care settings: study protocol. Implement Sci. 2008;3(1):15.

19.	Quanbeck A, Almirall D, Jacobson N, et al. The Balanced Opioid Initiative: protocol for a clustered, sequential, multiple-assignment randomized trial to construct an adaptive implementation strategy to improve guideline-concordant opioid prescribing in primary care. Implement Sci. 2020;15(1):26.

20.	Owen RR, Woodward EN, Drummond KL, et al. Using implementation facilitation to implement primary care mental health integration via clinical video telehealth in rural clinics: protocol for a hybrid type 2 cluster randomized stepped-wedge design. Implement Sci. 2019;14(1):33.

21.	Cucciare MA, Curran GM, Craske MG, et al. Assessing fidelity of cognitive behavioral therapy in rural VA clinics: design of a randomized implementation effectiveness (hybrid type III) trial. Implement Sci. 2016;11(1):65.

22.	Leonard C, Lawrence E, McCreight M, et al. Implementation and dissemination of a transition of care program for rural veterans: a controlled before and after study. Implement Sci. 2017;12(1):123.

23.	Hagedorn HJ, Brown R, Dawes M, et al. Enhancing access to alcohol use disorder pharmacotherapy and treatment in primary care settings: ADaPT-PC. Implement Sci. 2016;11(1):64.

24.	Parchman ML, Fagnan LJ, Dorr DA, et al. Study protocol for “Healthy Hearts Northwest”: a 2 × 2 randomized factorial trial to build quality improvement capacity in primary care. Implement Sci. 2016;11(1):138.

25.	Shelley DR, Ogedegbe G, Anane S, et al. Testing the use of practice facilitation in a cluster randomized stepped-wedge design trial to improve adherence to cardiovascular disease prevention guidelines: HealthyHearts NYC. Implement Sci. 2016;11(1):88.

26.	Becker SJ, DiClemente-Bosco K, Scott K, et al. Implementing contingency management for stimulant use in opioid treatment programs: protocol of a type III hybrid effectiveness-stepped-wedge trial. Implement Sci. 2023;18(1):41.

27.	Fox CH, Vest BM, Kahn LS, et al. Improving evidence-based primary care for chronic kidney disease: study protocol for a cluster randomized control trial for translating evidence into practice (TRANSLATE CKD). Implement Sci. 2013;8(1):88.

28.	Spoelstra SL, Schueller M, Sikorskii A. Testing an implementation strategy bundle on adoption and sustainability of evidence to optimize physical function in community-dwelling disabled and older adults in a Medicaid waiver: a multi-site pragmatic hybrid type III protocol. Implement Sci. 2019;14(1):60.

# Appendix C. Other Implementation Facilitation Resources

Additional implementation facilitation tools and resources described below can be found at: <https://www.queri.research.va.gov/tools/implementation.cfm>

**Implementation Facilitation Training Manual**: a comprehensive resource for individuals wanting to learn how to apply IF to enhance uptake and sustainment of evidence-based practices (EBPs) or other clinical innovations in their organization. The Manual: incorporates implementation science and clinical operations expertise; provides practical recommendations for improving EBP uptake by targeting barriers at multiple levels; and conveys IF research and theory as well as knowledge gained through real-world experiences of IF experts.

**Key Steps in Implementation Facilitation**: a 2-page addendum to the IF Training Manual that conveys concise, high-level information about the process for applying IF in 8 ‘Key Steps’ (i.e., core functions) to be used in most IF efforts.

**Implementation Facilitation Time and Activity Tracking Tool**: a templated Excel workbook used to document facilitators' time and activities (including core IF activities) for fidelity and cost analyses. The workbook includes instructions to implementation practitioners and researchers on how to adapt and customize the IF tracking tool for use in their own work.

**Implementation Facilitation Literature Collection**: a collection of peer-reviewed articles about application of IF to support adoption and sustained use of clinical innovations, including articles on research studies (e.g., RCTs), large-scale clinical initiatives, literature reviews, and conceptual/theoretical articles. The Collection can be used by healthcare providers and implementation practitioners to understand IF and how it has been applied in research studies and clinical initiatives. The Collection can also be used by researchers seeking to: understand theories/models/frameworks that have been used to guide IF strategies; develop plans to apply and evaluate IF strategies in their own research; and/or synthesize findings or lessons learned across multiple IF studies (e.g., in systematic reviews).
