# Supplementary material for: Dimensions of implementation facilitation (IF): development of a tool for designing, documenting and reporting on IF strategies in healthcare settings
Source: Implement Sci Commun. 2026 Jul 30;7:212. doi: 10.1186/s43058-026-01056-0 (PMC13711840; doi:10.1186/s43058-026-01056-0)
Supplement: Supplementary file 2 [file 43058_2026_1056_MOESM2_ESM.docx]

**Additional File 2. *Implementation Science* Protocol Paper Review data**

|  | **Position** | **Sources of influence** | **Activities** | **Duration** | **Interaction modes/ mediums** | **Frequency and intensity** | **Autonomy/ degree of flexibility** | **Role/ approach** | **Resources that support facilitator(s)** | **Change agents** | **IF skills, knowledge, characteristics** | **Other stakeholders** | **Self-efficacy** |
| --- | --- | --- | --- | --- | --- | --- | --- | --- | --- | --- | --- | --- | --- |
| Aifah 2021 | x | x | x | x | x | x |  |  | x | x | x | x |  |
| Aifah 2023 | x | x | x | x | x | x |  |  | x |  | x | x |  |
| Bahraini 2020 | x | x | x | x | x |  |  |  | x | x | x | x |  |
| Bauer 2016 | x | x | x | x | x | x | x | x |  |  |  | x |  |
| Becker 2023 | x |  | x | x | x | x |  |  | x |  |  | x |  |
| Beidas 2021 | x | x | x | x | x | x |  |  |  |  | x | x |  |
| Branch-Elliman 2022 | x |  | x |  | x | x |  |  | x |  | x | x |  |
| Bucknall 2017 | x | x | x | x | x | x | x | x | x | x | x |  |  |
| Campbell-Sherer 2014 | x | x | x | x |  |  |  |  |  | x |  | x |  |
| Carney 2019 |  |  | x |  |  |  | x |  |  |  |  | x |  |
| Chinman 2017 | x | x | x | x | x | x | x |  |  | x | x | x |  |
| Cucciare 2016 | x |  | x | x | x | x |  |  |  |  |  | x |  |
| Cully 2012 | x | x | x |  | x | x | x |  |  |  | x | x |  |
| Cumbe 2022 | x |  | x | x | x | x | x |  |  |  |  | x |  |
| DeVoe 2015 |  | x | x |  | x |  |  |  |  |  |  | x |  |
| D'Onofrio 2019 | x | x | x | x | x | x | x |  |  | x |  | x |  |
| Doran 2023 | x | x | x | x | x | x | x |  |  | x | x |  |  |
| Dorr 2015 |  |  | x | x |  | x | x |  |  |  |  |  |  |
| Eldh 2021 | x | x | x | x | x |  | x | x | x | x | x |  |  |
| Fasugba 2023 | x | x | x | x | x |  |  |  |  | x | x | x |  |
| Ford 2022 | x |  | x | x | x | x |  |  |  |  |  | x |  |
| Fox 2013 |  | x | x |  | x | x |  |  | x | x |  |  |  |
| Gabrielian 2022 | x | x | x | x | x | x |  |  |  |  | x | x |  |
| Geraci 2022 | x | x | x |  | x | x |  |  |  | x | x |  |  |
| Glass 2023 | x |  | x | x | x | x | x |  |  |  |  | x |  |
| Gold 2015 |  | x | x |  | x | x |  |  |  | x |  |  |  |
| Gold 2019 | x | x | x | x | x | x | x |  |  | x | x |  |  |
| Hagedorn 2016 | x | x | x | x | x | x |  |  |  | x |  |  |  |
| Harris 2013 | x | x | x | x | x | x |  |  | x |  | x | x |  |
| Hogue 2019 |  |  | x | x | x | x | x |  |  | x |  | x |  |
| Iverson 2020 | x | x | x | x | x | x |  |  | x |  | x | x |  |
| Kilbourne 2013 | x | x | x | x | x | x |  |  |  |  | x | x |  |
| Kilbourne 2014 | x | x | x | x | x | x |  |  |  |  | x | x |  |
| Kolko 2022 | x | x | x | x | x | x |  |  |  |  | x | x |  |
| Leonard 2017 | x | x | x |  | x | x | x | x | x | x | x |  |  |
| Liddy 2011 | x |  | x | x | x | x | x | x | x |  | x | x |  |
| Malone 2021 | x | x | x | x | x | x |  |  | x |  | x |  |  |
| Midboe 2018 | x | x | x | x | x | x |  |  | x | x | x | x |  |
| O'Grady 2021 | x |  | x | x |  | x | x |  |  | x |  | x |  |
| Owen 2013 | x | x | x | x |  |  | x |  |  | x | x |  |  |
| Owen 2019 | x | x | x | x |  |  | x |  |  | x | x | x |  |
| Parchman 2008 |  | x | x | x |  | x | x |  |  |  | x | x |  |
| Parchman 2016 | x | x | x | x | x | x |  | x | x |  |  | x |  |
| Quanbeck 2020 | x | x | x | x | x | x | x |  |  | x | x |  |  |
| Roberge 2013 | x | x | x | x | x | x | x | x | x | x | x | x |  |
| Seers 2012 | x |  | x | x |  |  |  | x | x |  | x | x |  |
| Shelley 2016 | x | x | x | x | x | x |  |  | x |  |  | x |  |
| Simmons 2017 | x | x | x | x |  |  |  |  | x |  | x | x |  |
| Spoelstra 2019 | x | x | x | x | x | x |  |  | x |  | x | x |  |
| Swindle 2022 | x | x | x | x |  | x | x | x | x | x | x | x |  |
| Tapp 2014 | x | x | x | x | x | x |  |  | x |  | x | x |  |
| Weiner 2015 | x |  |  | x |  | x |  |  |  |  |  |  |  |
| Yano 2016 | x | x | x |  | x | x |  |  |  | x |  |  |  |

**References**

Aifah AA, Odubela O, Rakhra A, Onakomaiya D, Hu J, Nwaozuru U, et al. Integration of a task strengthening strategy for hypertension management into HIV care in Nigeria: a cluster randomized controlled trial study protocol. Implement Sci. 2021;16(1):96. https://doi.org/10.1186/s13012-021-01167-3

Aifah AA, Hade EM, Colvin C, Henry D, Mishra S, Rakhra A, et al. Study design and protocol of a stepped wedge cluster randomized trial using a practical implementation strategy as a model for hypertension-HIV integration — the MAP-IT trial. Implement Sci. 2023;18(1):14. https://doi.org/10.1186/s13012-023-01272-5

Bahraini NH, Matarazzo BB, Barry CN, Post EP, Forster JE, Dollar KM, et al. Protocol: examining the effectiveness of an adaptive implementation intervention to improve uptake of the VA suicide risk identification strategy: a sequential multiple assignment randomized trial. Implement Sci. 2020;15(1):58. https://doi.org/10.1186/s13012-020-01019-6

Bauer MS, Miller C, Kim B, Lew R, Weaver K, Coldwell C, et al. Partnering with health system operations leadership to develop a controlled implementation trial. Implement Sci. 2016;11(1):22. https://doi.org/10.1186/s13012-016-0385-7

Becker SJ, DiClemente-Bosco K, Scott K, Janssen T, Salino SM, Hasan FN, et al. Implementing contingency management for stimulant use in opioid treatment programs: protocol of a type III hybrid effectiveness-stepped-wedge trial. Implement Sci. 2023;18(1):41. https://doi.org/10.1186/s13012-023-01297-w

Beidas RS, Ahmedani BK, Linn KA, Marcus SC, Johnson C, Maye M, et al. Study protocol for a type III hybrid effectiveness-implementation trial of strategies to implement firearm safety promotion as a universal suicide prevention strategy in pediatric primary care. Implement Sci. 2021;16(1):89. https://doi.org/10.1186/s13012-021-01154-8

Branch-Elliman W, Lamkin R, Shin M, Mull HJ, Epshtein I, Golenbock S, et al. Promoting de-implementation of inappropriate antimicrobial use in cardiac device procedures by expanding audit and feedback: protocol for hybrid III type effectiveness/implementation quasi-experimental study. Implement Sci. 2022;17(1):12. https://doi.org/10.1186/s13012-022-01186-8

Bucknall TK, Harvey G, Considine J, Mitchell I, Rycroft-Malone J, Graham ID, et al. Prioritising Responses Of Nurses To deteriorating patient Observations (PRONTO) protocol: testing the effectiveness of a facilitation intervention in a pragmatic, cluster-randomised trial with an embedded process evaluation and cost analysis. Implement Sci. 2017;12(1):85. https://doi.org/10.1186/s13012-017-0617-5

Campbell-Scherer DL, Asselin J, Osunlana AM, Fielding S, Anderson R, Rueda-Clausen CF, et al. Implementation and evaluation of the 5As framework of obesity management in primary care: design of the 5As Team (5AsT) randomized control trial. Implement Sci. 2014;9(1):78. https://doi.org/10.1186/1748-5908-9-78

Carney PA, Hatch B, Stock I, Dickinson C, Davis M, Larsen R, et al. A stepped-wedge cluster randomized trial designed to improve completion of HPV vaccine series and reduce missed opportunities to vaccinate in rural primary care practices. Implement Sci. 2019;14(1):30. https://doi.org/10.1186/s13012-019-0871-9

Chinman M, Daniels K, Smith J, McCarthy S, Medoff D, Peeples A, et al. Provision of peer specialist services in VA patient aligned care teams: protocol for testing a cluster randomized implementation trial. Implement Sci. 2017;12(1):57. https://doi.org/10.1186/s13012-017-0587-7

Cucciare MA, Curran GM, Craske MG, Abraham T, McCarthur MB, Marchant-Miros K, et al. Assessing fidelity of cognitive behavioral therapy in rural VA clinics: design of a randomized implementation effectiveness (hybrid type III) trial. Implement Sci. 2016;11(1):65. https://doi.org/10.1186/s13012-016-0432-4

Cully J, Armento ME, Mott J, Nadorff M, Naik A, Stanley M, et al. Brief cognitive behavioral therapy in primary care: A hybrid type 2 patient-randomized effectiveness-implementation design. Implement Sci. 2012;7(1):64. https://doi.org/10.1186/1748-5908-7-64

Cumbe VFJ, Muanido AG, Turner M, Ramiro I, Sherr K, Weiner BJ, et al. Systems analysis and improvement approach to optimize outpatient mental health treatment cascades in Mozambique (SAIA-MH): study protocol for a cluster randomized trial. Implement Sci. 2022;17(1):37.https://doi.org/10.1186/s13012-022-01213-8

DeVoe JE, Huguet N, Likumahuwa-Ackman S, Angier H, Nelson C, Marino M, et al. Testing health information technology tools to facilitate health insurance support: a protocol for an effectiveness-implementation hybrid randomized trial. Implement Sci. 2015;10(1):123. https://doi.org/10.1186/s13012-015-0311-4

D’Onofrio G, Edelman EJ, Hawk KF, Pantalon MV, Chawarski MC, Owens PH, et al. Implementation facilitation to promote emergency department-initiated buprenorphine for opioid use disorder: protocol for a hybrid type III effectiveness-implementation study (Project ED HEALTH). Implement Sci. 2019;14(1):48. https://doi.org/10.1186/s13012-019-0891-5

Doran KM, Torsiglieri A, Blaufarb S, Hernandez P, Melnick E, Velez L, et al. The POP (Permanent Supportive Housing Overdose Prevention) Study: protocol for a hybrid type 3 stepped-wedge cluster randomized controlled trial. Implement Sci. 2023;18(1):21. https://doi.org/10.1186/s13012-023-01278-z

Dorr DA, McConnell KJ, Williams MP-J, Gray KA, Wagner J, Fagnan LJ, et al. Study protocol: transforming outcomes for patients through medical home evaluation and redesign: a cluster randomized controlled trial to test high value elements for patient-centered medical homes versus quality improvement. Implement Sci. 2015;10(1):13. https://doi.org/10.1186/s13012-015-0204-6

Eldh AC, Joelsson-Alm E, Wretenberg P, Hälleberg-Nyman M. Onset PrevenTIon of urinary retention in Orthopaedic Nursing and rehabilitation, OPTION—a study protocol for a randomised trial by a multi-professional facilitator team and their first-line managers’ implementation strategy. Implement Sci. 2021;16(1):65. https://doi.org/10.1186/s13012-021-01135-x

Fasugba O, Dale S, McInnes E, Cadilhac DA, Noetel M, Coughlan K, et al. Evaluating remote facilitation intensity for multi-national translation of nurse-initiated stroke protocols (QASC Australasia): a protocol for a cluster randomised controlled trial. Implement Sci. 2023;18(1):2. https://doi.org/10.1186/s13012-023-01260-9

Ford JH, Cheng H, Gassman M, Fontaine H, Garneau HC, Keith R, et al. Stepped implementation-to-target: a study protocol of an adaptive trial to expand access to addiction medications. Implement Sci. 2022;17(1):64. https://doi.org/10.1186/s13012-022-01239-y

Fox CH, Vest BM, Kahn LS, Dickinson LM, Fang H, Pace W, et al. Improving evidence-based primary care for chronic kidney disease: study protocol for a cluster randomized control trial for translating evidence into practice (TRANSLATE CKD). Implement Sci. 2013;8(1):88. https://doi.org/10.1186/1748-5908-8-88

Gabrielian S, Finley EP, Ganz DA, Barnard JM, Jackson NJ, Montgomery AE, et al. Comparing two implementation strategies for implementing and sustaining a case management practice serving homeless-experienced veterans: a protocol for a type 3 hybrid cluster-randomized trial. Implement Sci. 2022;17(1):67. https://doi.org/10.1186/s13012-022-01236-1

Geraci JC, Finley EP, Edwards ER, Frankfurt S, Kurz AS, Kamdar N, et al. Partnered implementation of the veteran sponsorship initiative: protocol for a randomized hybrid type 2 effectiveness—implementation trial. Implement Sci. 2022;17(1):43. https://doi.org/10.1186/s13012-022-01212-9

Glass JE, Dorsey CN, Beatty T, Bobb JF, Wong ES, Palazzo L, et al. Study protocol for a factorial-randomized controlled trial evaluating the implementation, costs, effectiveness, and sustainment of digital therapeutics for substance use disorder in primary care (DIGITS Trial). Implement Sci. 2023;18(1):3. https://doi.org/10.1186/s13012-022-01258-9

Gold R, Hollombe C, Bunce A, Nelson C, Davis JV, Cowburn S, et al. Study protocol for “Study of Practices Enabling Implementation and Adaptation in the Safety Net (SPREAD-NET)”: a pragmatic trial comparing implementation strategies. Implement Sci. 2015;10(1):144. https://doi.org/10.1186/s13012-015-0333-y

Gold R, Bunce A, Cottrell E, Marino M, Middendorf M, Cowburn S, et al. Study protocol: a pragmatic, stepped-wedge trial of tailored support for implementing social determinants of health documentation/action in community health centers, with realist evaluation. Implement Sci. 2019;14(1):9. https://doi.org/10.1186/s13012-019-0855-9

Hagedorn HJ, Brown R, Dawes M, Dieperink E, Myrick DH, Oliva EM, et al. Enhancing access to alcohol use disorder pharmacotherapy and treatment in primary care settings: ADaPT-PC. Implement Sci. 2016;11(1):64. https://doi.org/10.1186/s13012-016-0431-5

Harris MF, Lloyd J, Litt J, van Driel M, Mazza D, Russell G, et al. Preventive evidence into practice (PEP) study: implementation of guidelines to prevent primary vascular disease in general practice protocol for a cluster randomised controlled trial. Implement Sci. 2013;8(1):8. https://doi.org/10.1186/1748-5908-8-8

Hogue A, Dauber S, Bobek M, Jensen-Doss A, Henderson CE. Measurement Training and Feedback System for Implementation of family-based services for adolescent substance use: protocol for a cluster randomized trial of two implementation strategies. Implement Sci. 2019;14(1):25. https://doi.org/10.1186/s13012-019-0874-6

Iverson KM, Dichter ME, Stolzmann K, Adjognon OL, Lew RA, Bruce LE, et al. Assessing the Veterans Health Administration’s response to intimate partner violence among women: protocol for a randomized hybrid type 2 implementation-effectiveness trial. Implement Sci. 2020;15(1):29. https://doi.org/10.1186/s13012-020-0969-0

Kilbourne AM, Abraham K, Goodrich D, Bowersox N, Almirall D, Lai Z, et al. Cluster randomized adaptive implementation trial comparing a standard versus enhanced implementation intervention to improve uptake of an effective re-engagement program for patients with serious mental illness. Implement Sci. 2013;8(1):136. https://doi.org/10.1186/1748-5908-8-136

Kilbourne AM, Almirall D, Eisenberg D, Waxmonsky J, Goodrich DE, Fortney JC, et al. Protocol: Adaptive Implementation of Effective Programs Trial (ADEPT): cluster randomized SMART trial comparing a standard versus enhanced implementation strategy to improve outcomes of a mood disorders program. Implement Sci. 2014;9:132. https://doi.org/10.1186/s13012-014-0132-x

Kolko DJ, McGuier EA, Turchi R, Thompson E, Iyengar S, Smith SN, et al. Care team and practice-level implementation strategies to optimize pediatric collaborative care: study protocol for a cluster-randomized hybrid type III trial. Implement Sci. 2022;17(1):20. https://doi.org/10.1186/s13012-022-01195-7

Leonard C, Lawrence E, McCreight M, Lippmann B, Kelley L, Mayberry A, et al. Implementation and dissemination of a transition of care program for rural veterans: a controlled before and after study. Implement Sci. 2017;12(1):123. https://doi.org/10.1186/s13012-017-0653-1

Liddy C, Hogg W, Russell G, Wells G, Armstrong CD, Akbari A, et al. Improved delivery of cardiovascular care (IDOCC) through outreach facilitation: study protocol and implementation details of a cluster randomized controlled trial in primary care. Implement Sci. 2011;6(1):110. https://doi.org/10.1186/1748-5908-6-110

Malone S, McKay VR, Krucylak C, Powell BJ, Liu J, Terrill C, et al. A cluster randomized stepped-wedge trial to de-implement unnecessary post-operative antibiotics in children: the optimizing perioperative antibiotic in children (OPerAtiC) trial. Implement Sci. 2021;16(1):29. https://doi.org/10.1186/s13012-021-01096-1

Midboe AM, Martino S, Krein SL, Frank JW, Painter JT, Chandler M, et al. Testing implementation facilitation of a primary care-based collaborative care clinical program using a hybrid type III interrupted time series design: a study protocol. Implement Sci. 2018;13(1):145. https://doi.org/10.1186/s13012-018-0838-2

O’Grady MA, Lincourt P, Greenfield B, Manseau MW, Hussain S, Genece KG, et al. A facilitation model for implementing quality improvement practices to enhance outpatient substance use disorder treatment outcomes: a stepped-wedge randomized controlled trial study protocol. Implement Sci. 2021;16(1):5. https://doi.org/10.1186/s13012-020-01076-x

Owen R, Drummond K, Viverito K, Marchant K, Pope S, Smith J, et al. Monitoring and managing metabolic effects of antipsychotics: a cluster randomized trial of an intervention combining evidence-based quality improvement and external facilitation. Implement Sci. 2013;8(1):120. https://doi.org/10.1186/1748-5908-8-120

Owen RR, Woodward EN, Drummond KL, Deen TL, Oliver KA, Petersen NJ, et al. Using implementation facilitation to implement primary care mental health integration via clinical video telehealth in rural clinics: protocol for a hybrid type 2 cluster randomized stepped-wedge design. Implement Sci. 2019;14(1):33. https://doi.org/10.1186/s13012-019-0875-5

Parchman ML, Pugh JA, Culler SD, Noel PH, Arar NH, Romero RL, et al. A group randomized trial of a complexity-based organizational intervention to improve risk factors for diabetes complications in primary care settings: study protocol. Implement Sci. 2008;3(1):15. https://doi.org/10.1186/1748-5908-3-15

Parchman ML, Fagnan LJ, Dorr DA, Evans P, Cook AJ, Penfold RB, et al. Study protocol for “Healthy Hearts Northwest”: a 2 × 2 randomized factorial trial to build quality improvement capacity in primary care. Implement Sci. 2016;11(1):138. https://doi.org/10.1186/s13012-016-0502-7

Quanbeck A, Almirall D, Jacobson N, Brown RT, Landeck JK, Madden L, et al. The Balanced Opioid Initiative: protocol for a clustered, sequential, multiple-assignment randomized trial to construct an adaptive implementation strategy to improve guideline-concordant opioid prescribing in primary care. Implement Sci. 2020;15(1):26. https://doi.org/10.1186/s13012-020-00990-4

Roberge P, Fournier L, Brouillet H, Hudon C, Houle J, Provencher MD, et al. Implementing a knowledge application program for anxiety and depression in community-based primary mental health care: a multiple case study research protocol. Implement Sci. 2013;8(1):26. https://doi.org/10.1186/1748-5908-8-26

Seers K, Cox K, Crichton NJ, Edwards RT, Eldh AC, Estabrooks CA, et al. FIRE (facilitating implementation of research evidence): a study protocol. Implement Sci. 2012;7(1):25. http://dx.doi.org/10.1186/1748-5908-7-25

Shelley DR, Ogedegbe G, Anane S, Wu WY, Goldfeld K, Gold HT, et al. Testing the use of practice facilitation in a cluster randomized stepped-wedge design trial to improve adherence to cardiovascular disease prevention guidelines: HealthyHearts NYC. Implement Sci. 2016;11(1):88. https://doi.org/10.1186/s13012-016-0450-2

Simmons MM, Gabrielian S, Byrne T, McCullough MB, Smith JL, Taylor TJ, et al. A Hybrid III stepped wedge cluster randomized trial testing an implementation strategy to facilitate the use of an evidence-based practice in VA Homeless Primary Care Treatment Programs. Implement Sci. 2017;12(1):46. https://doi.org/10.1186/s13012-017-0563-2

Spoelstra SL, Schueller M, Sikorskii A. Testing an implementation strategy bundle on adoption and sustainability of evidence to optimize physical function in community-dwelling disabled and older adults in a Medicaid waiver: a multi-site pragmatic hybrid type III protocol. Implement Sci. 2019;14(1):60. https://doi.org/10.1186/s13012-019-0907-1

Swindle T, Rutledge JM, Selig JP, Painter J, Zhang D, Martin J, et al. Obesity prevention practices in early care and education settings: an adaptive implementation trial. Implement Sci. 2022;17(1):25. https://doi.org/10.1186/s13012-021-01185-1

Tapp H, McWilliams A, Ludden T, Kuhn L, Taylor Y, Alkhazraji T, et al. Comparing traditional and participatory dissemination of a shared decision making intervention (ADAPT-NC): a cluster randomized trial. Implement Sci. 2014;9(1):158. https://doi.org/10.1186/s13012-014-0158-0

Weiner BJ, Pignone MP, DuBard CA, Lefebvre A, Suttie JL, Freburger JK, et al. Advancing heart health in North Carolina primary care: the Heart Health NOW study protocol. Implement Sci. 2015;10(1):160. https://doi.org/10.1186/s13012-015-0348-4

Yano EM, Darling JE, Hamilton AB, Canelo I, Chuang E, Meredith LS, et al. Cluster randomized trial of a multilevel evidence-based quality improvement approach to tailoring VA Patient Aligned Care Teams to the needs of women Veterans. Implement Sci. 2016;11(1):101. https://doi.org/10.1186/s13012-016-0461-z
